# Supplementary material for: Early-career setback and future career impact
Source: Nat Commun. 2019 Oct 1;10:4331. doi: 10.1038/s41467-019-12189-3 (PMC6773762; doi:10.1038/s41467-019-12189-3)
Supplement: Supplementary file 1 — Supplementary Information [file 41467_2019_12189_MOESM1_ESM.pdf]

Early-career setback and future career impact

Wang et al.

Table of Contents

Supplementary Note 1: Data Description 3
Supplementary Note 2: Related Work 9
Supplementary Note 3: Robustness Checks and Additional Results 11
Supplementary Note 4: Potential generative processes 23
Supplementary Figures 28
Supplementary References 59

Supplementary Note 1: Data Description

In this paper, we assembled large-scale datasets from a range of different sources: 1) the U.S. National Institutes of Health (NIH) grant application data; 2) Clarivate Analytics' Web of Science data (WoS); 3) the National Library of Medicine's PubMed database; and 4) Google Scholar (GS) profiles. Combining these datasets allows us to trace career trajectories of NIH principal investigators (PIs) through their grant applications, funding outcomes, scientific publications and publication impacts.

NIH grant database and sample details

Our main dataset contains all R01 grant applications ever submitted to the NIH between FY1985 and FY2015; R01 is the primary funding mechanism NIH, which is the world's largest funder for biomedical research. Our data consist of 778,219 competing grant applications in total, supporting more than 170,000 research personnel across more than 2,500 U.S. institutions. For each grant application, we obtained its evaluation score, scientific study section, a unique identifier for the PI, the PI's name, and the funding outcome. Supplementary Figure 1 shows the number of competing R01 applications and the success rate over time; the success rate has declined over the past three decades, due to the increased number of applications with limited budgets to support them (Supplementary Figure 1b).

Publication and citation datasets

Our second dataset, i.e., the Web of Science, provides comprehensive publication and citation records of more than 46 million papers published from 1900 to 2015 by more than 20,000 different journals. For each paper, the WoS data contain the title, journal, subject, publication date, author names, affiliations, and a set of references.

For every NIH grant, all papers published as a result of the grant are deposited in the PubMed database and uniquely identified with PubMed IDs. This fact enables two important possibilities. First, it allows us to link the NIH grant database with the WoS dataset using a precise mapping between PubMed IDs and paper identifiers in our WoS database purchased through an advanced agreement with Clarivate Analytics. This mapping not only offers us additional information about

each publication, it also allows us to trace citations to each paper within the WoS and how these citations compare with other papers published in the same year and field. Second, each PI is associated with his/her unique IDs in the NIH data, and the PubMed papers associated with all his/her grants offer a set of "ground-truth" papers for disambiguation purposes. As such this key feature of our data could also substantially improve the accuracy with which we identify authors in the publication databases, as we will discuss in detail later.

NIH grant review process

To make sure all readers are familiar with the process through which our study took place, here we briefly review the NIH grant review process[1]: In the first step, each PI submits his/her grant proposal directly to the Center for Scientific Review (CSR), and a referral officer will assign the application to an appropriate Integrated Review Group (IRG), Scientific Review Group (SRG) and Institute or Center (I/C). After assignment, each application receives an identification number. All applications will be assigned to a review group (known as a study section) that comprises scientific peers, where reviewers usually evaluate the proposal according to several metrics such as innovativeness, significance, investigator's track record, et cetera. About 50% of all applications are rejected prior to the panel discussion step, and thus no score is assigned. For applications that reach the discussion panel, each study section member provides a priority score from 1.0 (best) to 5.0 (worst) with increments of 0.1, and the score is averaged and multiplied by a factor of 100. These scores are then normalized within each study section to facilitate funding decisions.

In this study, we focus on all new R01 competing grant applications from regular standing study sections between FY1990 and FY2005. We did not use data from FY1985 to FY1989 to eliminate possible boundary effects in defining junior PIs. Similarly, we focus on grants prior to FY2006 because we need additional ten-year time window to trace career outcomes. To further make sure that near-miss and narrow-win individuals studied in our paper apply to categorically similar R01 programs, we eliminated special-emphasis panels (special emphasis panels are review groups formed on an ad hoc basis to review applications requiring special expertise or when a conflict of interest situation occurs), as well as small study sections with less than 50 grant applications per year, to ensure statistical power when making inferences about proposals close to the payline.

Author name disambiguation

Each PI in the NIH is uniquely identified by an NIH ID together with his or her full name, and all publications resulting from his/her NIH grants are correctly associated with the PI. This feature provides useful information that helps with author name disambiguation. For any paper in a scholarly database, a fundamental issue is to identify the individual(s) who wrote it and, conversely, to identify all of the works that belong to a given individual[2-4]. This seemingly simple task represents a major unsolved problem for information and computer sciences, mainly because of the lack of ground truth. While automatic name disambiguation in large-scale scholarly datasets remains an unsolved challenge, recent studies on individual careers have achieved initial success using various pertinent citation features[2,5]. In this paper, we build on the state-of-art algorithms but also extends them in meaningful ways by combining the NIH dataset with the WoS dataset. Indeed, the NIH publications associated with each PI represent new, ground-truth information that none of the existing disambiguation algorithms had access to. When working in unison with existing algorithms, such information allows us to further harvest additional publications by the PI, which could potentially offer a more accurate and comprehensive trace for individual career histories than simply using the state-of-art algorithms. Next we describe specific steps in our name disambiguation procedure.

First, for each PI name we generated a pool of candidate publications, consisting of all possible publications by the PI from the WoS according to the following rules: a) Last names of these publications must be the same with the PI last name; b) Initials of the first names have to be the same with the first name of this PI. If full first names from the WoS are available, they must be identical to this PI's first name; c) The same rule applies to middle names. One can think of the goal of this step as to increase the rate of recall at the expense of precision.

Our second step involves merging papers from the pool generated above. Here we build on existing algorithms[2,6,7] to decide the authorship of papers: Two papers are considered to be from the same author if one of the following conditions is true: a) The two papers cite each other; b) The two papers share at least one common reference.

After step two, we compare papers from each group with NIH supported publications by the considered PI, following these two rules: a) If the PI has at least one NIH-related publications, all groups that contain these publications are associated with this PI; b) If there is no NIH-related publication for a particular PI, we compared the string similarity between PI's affiliation on the NIH grant applications and on the publications, after removing common words (e.g., stop words, "university", "institute", "hospital", etc.), and consider the paper belongs to the PI for the cases with high cosine similarity (in our case, we set the threshold at 0.65), as well as its group.

Although name disambiguation methods are becoming increasingly accurate, the main problem with them is that we are often not sure how perfect or imperfect they are. To gauge the accuracy of our algorithm, we compare career histories obtained using our method with Google Scholar (GS) individual profiles. GS allows individual scientists to create, maintain, and update their own profile of publication records, assisted by the name disambiguation algorithm developed by Google. While the GS profiles themselves are not gold standards, here we quantify the extent of agreement between our method and GS to make sure that our name disambiguation method does not deviate significantly from an independent test. Comparing with GS profiles, we find that the average precision and recall of our algorithm is 0.85, and 0.71, respectively. An alternative way that we could make use of the GS profiles is to incorporate them directly into our disambiguation procedure. The reason we did not do that is to avoid potential bias in our results, because not everyone in our sample has a GS profile. Indeed, we find that only a fraction of NIH PIs has reliable GS profiles, which are biased towards active PIs. Since the goal of our study involves testing differences between two populations, the potential bias introduced by the GS profiles could be an issue as more narrow wins remained active in our setting.

To further evaluate the accuracy of our algorithm to disambiguate author names, we randomly select 1,000 author-publication pairs from PIs around the payline. Specifically, we randomly select 50 PIs, and for each PI 20 publications. By removing meeting abstracts in the Web of Science data, we obtained 576 positive cases in which papers were published by the PI, and 225 negative cases in which papers were written by different persons with similar name as the PI. We then performed manually search for PIs' websites, Google Scholar profiles if any, affiliations, coauthors etc. Out of these 801 pairs, we find the false positive rate (i.e. fraction of times the algorithm indicates the

paper belonging to the PI, while they do not) to be 8.9% and a false negative rate (i.e. fraction of times that the algorithm considers the paper belonging to a different person, while they do not) of 4.7%. These low error rates support the validity of the disambiguation algorithm we used in the main text.

The analyses in the preceding paragraph show a high degree of agreement between our method and an independent test set; the method also show low error rate with respect to manually searches. As with any name disambiguation methods, it is necessarily imperfect. To ensure the errors contained in the method do not affect the conclusions of our study, and to examine further the robustness of our results, we repeated our analyses by varying different assumptions made in the disambiguation procedure (Supplementary Figure 2). For each variation, we redo the disambiguation process and repeat our analyses to see if the conclusions change. For example, in Method A, we considered only self-citations between two papers to determine if they are from the same author; In Method B, we used not only self-citations and co-references, but also take into account the names of coauthors. For example, if two papers associated to the same name share at least one common coauthor name, or one common reference, or cite each other, they are considered to be from the same PI; In Method C, we implemented the name disambiguation method proposed by Caron and van Eck[8,9]. The method takes other information into consideration including common citations, common addresses, email, journal and subject categories, etc. Two papers are clustered if the score exceeds a certain threshold. These different methods result in different set of papers for each author. Yet we find, amid all different variations, our conclusions remain the same (Supplementary Figure 2). This supports the hypothesis that while name disambiguation methods may be error-prone, the errors do not affect the conclusion if they are distributed unbiased across the two populations. Indeed, we find that, under different disambiguation methods, near misses consistently have a higher chance to publish hit papers (Supplementary Figure 2a, d, and g). Near misses also outperform narrow wins in terms of the average number of citations per paper (Supplementary Figure 2b, e, and h). In terms of the number of publications, as in the main text there is again a lack of significant difference between the two groups (Supplementary Figure 2c, f, and i, Figure 2a in the main text). Finally, PIs with common Chinese or Korean names are known to be difficult to disambiguate, accounting for the majority of the error rate[2,3,10]. As another

robustness check, we repeated our analyses by removing PIs with common Chinese or Korean last names (Supplementary Figure 3), finding the results are similar to those reported in the main text.

Although name disambiguation methods are becoming increasingly accurate, the main problem is that we often don't know how perfect or imperfect they are. Yet, while the name disambiguation methods may be imperfect, it's important to note that there are also reasons to believe that potential errors in name disambiguation methods do not affect our conclusions, as such errors are likely to differ smoothly with the score hence are controlled for by our RD analyses. In other words, the errors don't favor any particular group above or below the payline. Nevertheless, our goal here is to try several state-of-art name disambiguation methods to further ensure the robustness of our results (Supplementary Figure 2 and Supplementary Figure 3).

Normalized Priority Score

In this study, we take advantage of the fact that NIH funding decisions are based on evaluation scores, resulting in a highly non-linear relationship with funding success. The funding cutoff at the NIH varies across different study sections, institutes, year and grant mechanisms. To infer the cutoff, here we build on prior work[11] to obtain the priority score for each application. In addition, our data allowed us to zoom into each study section instead of focusing on the NIH institute level. This helps us account for the heterogeneity of cutoff across different years and study sections, by comparing, for each fiscal year, the grant applications submitted to the same study section. We investigated the success probability as a function of priority score and define the cutoff score as the third worst score of funded applications in each study section so that there are only two out-of-order funding events that occurred beyond the cutoff. This choice helps balance the number of observations in both narrow-win and near-miss group. To check if it indeed leads to a cutoff effect, we calculate the normalized score by subtracting the cutoff score from each proposal score, finding a clear drop in funding success probability at the cutoff point (Supplementary Figure 4 and Figure 1a); the same results hold for major NIH institutes (Supplementary Figure 4b). We also repeated our analyses by defining the cutoff as the second worst score of funded applications in each study section, finding consistent conclusions (Supplementary Figure 5).

Supplementary Note 2: Related Work

There has been extant theoretical and empirical evidence from a diverse set of disciplines that can help inform our research question of whether an early-career setback may lead to future career impact. Here we aim to provide a brief overview of the different lines of research.

An especially prominent and relevant line of enquiry is the Matthew effect, which was formalized by Robert K. Merton in 1968[12], named after a verse in the bible's Gospel of Matthew. Merton described a "rich get richer" phenomenon, where early-career success brings reputation and recognition that can translate into tangible assets that in turn help bring future successes. The idea of the Matthew effect has deep roots, and goes under many different titles, including cumulative advantage[13] used by de Solla Price to explain citations of papers, and preferential attachment[14] in the network science context to explain the origin of scale-free networks. The idea also forms the core of various fundamental, mathematical models, including Pólya process, corresponding to the well-known urn model by György Pólya in 1923, or the Yule process[15]. It also underlies the key assumption used by Zipf to explain the fat tailed distribution of wealth in the society[16].

The Matthew effect predicts that the answer to the question of whether early setbacks lead to longer-term success should be no. This thesis is further supported by widespread empirical evidence in science as well as in other related domains. For example, for publications, the Matthew effect captures the well-documented fact that highly-cited papers are more visible and are more likely to be cited again in the future[13,17-21]. Studies of individual scientific careers documented the reputation effect that an early-career recognition brings to increase future chances of success[22-26]. Beyond science, the Matthew effect has also been experimentally validated in various domains, from social influence[27] to different reward systems[28,29]. It also speaks to a classical line of research that success may relate to the sacred spark[30,31] or influence individual motivation, where positive feedback bolsters self-confidence whereas failure lowers self-esteem.

The strongest endorsement to the Matthew effect comes from the fact that it has been investigated specifically in the setting of our study over the past decade[11,32,33]. For example, Jacob and Lefgren first documented the Matthew effect in the science funding system[11]. Using data from the NIH,

they found that a small but significant impact of initial NIH funding on obtaining future funding from the NIH, with little impact on future scientific productivity (i.e., number of publications). Ganguli investigated the impact of grants using data from a Soviet grant program shortly after the end of the USSR[32]. Her analysis directly speaks to the scenario when funding levels are low, and found that obtaining grants significantly reduces the attrition rate while increases future productivity. The most recent evidence of the Matthew effect in science funding was offered in the context of early-career academic funding system in the Netherlands[33]. More specifically, Bol et al. demonstrated that early career funding is critical for late-career funding, as winners just above the funding threshold accumulated more than twice as much as funding in the following eight years. Although in the Dutch setting, prior success of getting funded is a merit review criterion in later competitions which by itself may increase future chance for funding, these results are consistent with another recent study on the effect of postdoctoral fellowships[34]. where researchers showed that securing a specific postdoctoral fellowship from the NIH increases subsequent chances of getting another NIH grant. Together, the consistent results across all these studies offer clear, convincing endorsement that establishes the Matthew effect as the leading hypothesis for the research question raised in our paper. These studies are what make our findings surprising, thus highlighting the main novelty of our paper.

While the Matthew effect predicts that success breeds success, there are reasons to believe that the opposite may be true. The primary mechanism for this second school of thought is the screening effect, which builds on the observation that later competitions are held only among those who managed to stay in. Hence if early career setbacks increase attrition, it may act as a screening mechanism, leading to a more selected set of individuals who remain[35,36], with fixed, advantageous characteristics such as commitment, grit, high self-confidence, etc. As such, past failure may become a marker for future success for those who managed to stay in. This screening mechanism may produce empirically similar observations as the learning-from-failure literature, suggesting that failure may teach valuable lessons that is hard to learn otherwise[37-39]. In a separate line within the motivation literature, it has been shown that setbacks may also motivate people by signaling that more effort is needed, whereas success may associate with reduced future effort due to utility maximization[40] or offer a sense of partial goal attainment, signaling that less effort is needed to reach similar targets[40-42].

Finally, it is important to note that, while these different schools of thought make opposite predictions, they are not exclusive to each other. Rather they may both operate at the same time. Therefore, the net effect, which is what we can observe from data, remains unclear; this is the main purpose of our study.

Supplementary Note 3: Robustness Checks and Additional Results

In this section, we reported further robustness checks and additional results by constructing different experimental settings and parameters.

Hits per capita

In this section, we compared near misses with narrow wins in terms of hits per capita, measuring the average number of hit papers per person. We find a similar size of performance increases for near misses (near misses outperformed narrow wins by a factor of 19.2%, i.e., around 2 hit papers per capita over the next ten-year time window). The lesser significance level is due to a reduced sample size (Supplementary Figure 8, *t*-test, *p*-value = 0.107). Moreover, the RD analyses yields similar magnitude of results (a single near miss leads to 3.6 more hit papers per capita over the next ten-year time window, *p*-value 0.098, Supplementary Figure 7c).

Alternative definitions of junior PIs

In the main text, we defined junior PIs as those within the first three years of their R01 NIH applications. To ensure our results are not affected by this definition, here we tried two variations of alternative variations and repeat all our analyses. First, we modified our definition of junior PIs by only focusing on first-time applicants, i.e., those who submitted their first R01 application at the time of treatment. This results in a corpus of 656 narrows wins and 703 near misses. We conducted the same analyses, finding the results are robust across various measures of longer-term success (Supplementary Figure 9).

We also varied the junior PI definition by focusing on those without any existing NIH grants at the moment of treatment within our main sample. We recovered the same findings for this group of PIs. For those who managed to stay in, their subsequent publications again garnered higher impacts in terms of probability of producing a hit paper (Supplementary Figure 10a), average citations (Supplementary Figure 10b), or hit papers per capita (Supplementary Figure 10c). To test the screening effect, we repeated our conservative removal procedure, finding again that near misses significantly outperformed narrow wins (Supplementary Figure 10d-f), with a stronger effect than what we observed in Figure 3 of the main text (shaded symbols in Supplementary Figure 10d-f). Together these results indicate that our results are robust against different definitions of junior PIs.

Varying thresholds for the definitions of hit papers

To test if our results only hold for publications that fall within the top 5% of citations, we varied the definition of hit papers using top 1%, top 10% and top 15% of citations received in the same field (as indicated by the Web of Science subject categories) and year (Supplementary Figure 11). Consistent with our main results, both narrow wins and near misses have significantly higher hit paper probabilities relative to the base rate (i.e., 5%), independent of our definitions. In all three different definitions, near misses outperformed narrow wins, demonstrating that the hit rate per paper by the near misses are significantly higher than that of narrow wins. In terms of hits per capita, near misses published significantly more top 1% highly cited papers than narrow wins. Using our conservative removal method, we arrived at the same conclusion that the screening mechanism alone seems insufficient to explain the observed difference.

Normalized citations over time and disciplines

Publications in some disciplines may be cited more frequently than in other disciplines[43]. To normalize the raw citations with respect to different disciplines, we follow the canonical method[43] and normalize the citations of each article by the average citations of all articles belonging to the same field and year. Specifically, let the raw citation of our focus article be c, and the mean citations for all articles in the same field (as indicated by the Web of Science subject categories) and year be c_0. Then the normalized citations can be calculated as $c_f = c/c_0$. Moreover, we define

field according to the citation network of each article. By dividing the average citation rates by an expected citation rate from papers in the same field as indicated by the co-citation network, we calculated the relative citation ratio (RCR) for each article[44,45].

To show our results are robust with different citation measurements, we compare the average c_f and RCR of articles published by narrow wins and near misses (Supplementary Figure 12). We find that near misses again outperformed narrow wins in terms of the normalized citations and the RCR. Both groups have substantially higher citations compared with the average citations of all articles in the same field and same year. In the first five years after treatment, near misses attracted 15% more citations compared with narrow wins, and this difference persisted for the next five years. Consistent with our main finding, the screening effect seems insufficient to account for the entire difference (Supplementary Figure 12b, d).

Robustness to alternative fiscal years

To rule out the possibility that temporal effects drive the differences between narrow wins and near misses, in this section we focus on two sub-periods to see if our results hold in both time windows. We focus on two different time spans, from 1990 to 1995 and from 1995 to 2000. We find the results are robust with respect to different time periods (Supplementary Figure 14): in terms of hit rate per paper and average citations, near misses significantly outperformed narrow wins; in terms of hits per capita, we obtain the same direction with lesser significance level. In terms of the number of publications, there is no significant difference.

Additional funding by near misses

While NIH is the world's largest public funder for biomedical research, there may still exist the possibility that near-miss PIs found funding elsewhere. In this section, we extensively searched for other funding sources for near misses. Our procedure is as follow:

First, we searched for the names and affiliation information for all PIs through google to see whether there is any homepage that shows up in the first page of the search. Among all the searches, we find 66% returned official homepages (i.e., university faculty pages that contain mostly

biographic information only). However, we find that only 2.8% searches resulted in personal CVs, showing a very limited coverage via direct searches.

Given this limited coverage, we further drilled down on 200 PIs for detailed screening, and investigated each of them manually. Specifically, we randomly selected 100 scientists from the narrow-win group, 100 from the near-miss group, and manually searched for their profiles (including lab websites, personal websites, personal CVs etc.). After an extensive search process, our efforts yielded 13 (of 100) observations of CVs or personal website listing prior funding information for the near misses, and 10 (of 100) for the narrow wins; such sample size indicates that it is unlikely to yield meaningful statistics to compare the two groups. Although collecting CVs or personal websites didn't quite work out, to overcome this roadblock, we further tried *three* new approaches. We next describe these approaches in detail.

Approach A: acquired a novel data capturing individual funding history

Our first attempt is to search for large-scale datasets that can capture individual grant records, which led us to discover a new data source that seems ideal for our purpose: The Dimensions data, a major new data product from the Digital Science. It specializes in collecting funding histories for each individual scientist by assembling and integrating all publicly available funding records from all agencies around the world. And it contains more than 4.8 million funding records funded by 340 funding agencies from 40 countries and represents to our knowledge the most authoritative data source for this purpose.

For each PI in our sample, we manually searched for scientists with the same name in the same period, and retrieved his/her grant history recorded in the data using the online interface on Dimensions. First, the results show that narrow wins got significantly more NIH funding within 5 years after treatment (*t*-test, *p*-value = 0.04), but not for year 6 to year 10 (*t*-test, *p*-value = 0.54). The results are in consistent with the figures we reported in the manuscript (Figure 2d), suggesting the validity of the data. Also, narrow wins also obtained more NSF funding in the next five-year window (*t*-test, *p*-value = 0.02, Supplementary Figure 15), but not for year 6 to year 10 (*t*-test, *p*-value = 0.73). The results so far indicate that it is the narrow-win group who got more funding

within 5 years after treatment, suggesting the Matthew effect in science. We then measured the funding amount received by each PI from agencies other than NIH or NSF in the ten-year window following the treatment. We find the median funding amount per person for near misses between year 1 to year 5 is $370,000, and $610,000 for narrow wins, showing marginal significant difference between the two group with narrow wins having slightly more funding on average (*t*-test, *p*-value = 0.07). We then measured the funding amount for the two groups between year 6 to year 10, obtaining $670,000 and $600,000 per person for near misses and narrow wins, respectively (*t*-test, *p*-value = 0.34). We also find narrow wins obtained slightly more funding when comparing the two groups between year 1 to year 10 (*t*-test, *p*-value = 0.08). Together these results demonstrate that in the ten-year period, near misses did not acquire more funding from agencies other than NIH or NSF, compared with narrow wins.

While the approach above offers large-scale, quantitative evidence for this question, we further performed two additional approaches that offer further support for this conclusion:

Approach B: collecting funding acknowledgement from PubMed data

We also looked into the PubMed acknowledgement data, which contains research grants supported by any agency of the United States Public Health Service from 1981 (e.g., NIH, CDC, FDA, etc.). After 2005, the dataset also includes grant information for many other US or non-US funding agencies and organizations. Though we do not have funding amount in this dataset, we find that there is a lack of difference between near misses and narrow wins in the number of agencies other than the NIH or NSF. Specifically, within 5 years after treatment there were on average 0.80 funding agencies other than the NIH or NSF per person for narrow wins; for near misses, this number is 0.81 (*t*-test, *p*-value = 0.92). Between year 6 to year 10, there were on average 2.03 other funding agencies per person for narrow wins; for near misses, this number is 1.50 (*t*-test, *p*-value = 0.40)

Approach C: manually checking acknowledge statements included in publications

To further make sure our results are not affected by the PubMed coverage, we manually checked the acknowledgement statement of 100 random selected papers within 5 years after the treatment (50 for each group). We downloaded the PDFs, and read through them one by one. We arrived at the same conclusions (0.47 other grants per paper for narrow wins, 0.35 other grants per paper for near misses, t-test, p-value = 0.45).

In conclusion, our analyses provide empirical evidence that near misses were unlikely to receive more funding from agencies other than NIH or NSF.

Publication lags

In this section, we conduct additional analyses to check whether the results reported in the main text are affected by publication lags where near misses had many papers unpublished at the time of treatment but under the peer review process. Given that the median time of a manuscript from submission to acceptance in a journal is around half a year[46], we took papers published within one year after treatment in case they went for different journals. We compared the hit probability for near misses and narrow wins for year 0 and year 1, finding there is a lack of difference in the hit rate between near misses and narrow wins (14.9% for near misses versus 15.3% for narrow wins, χ^2-test, p-value = 0.50). This result is consistent with our RD design, indicating that the two groups should be similar across various characteristics *ex ante*, including the characteristics of works in the pipeline. It also appears to reject the hypothesis that the observed difference between the two groups is simply due to hit papers moving through the pipeline.

Building on this analysis, we next investigate where hit papers occurred within the subsequent 10-year period. We divided the 10-year window into two non-overlapping periods; specifically, from 1 to 3 years and from 4 to 10 years. If the pipeline hypothesis is correct, then hit papers by the near misses should be more concentrated in the initial period. However, when we break our data into these two periods, we find that hit papers by near misses were not concentrated in the initial period following the treatment (Supplementary Figure 16). Rather, the effect tends to be much more pronounced from year 4 to 10.

Together these results show that the performance advantage by the near misses did not occur overnight, suggesting that publication lags are unlikely to explain why near misses outperformed narrow wins.

Different field definition

In this section, we collected Medical Subject Headings (MeSH) using the PubMed dataset, which is a hierarchically-organized terminology for indexing and cataloging biomedical information. The goal here is to use the MeSH terms to define fields and then repeat our analyses to see if our results still hold. More specifically, for each paper in the PubMed database, there are multiple MeSH terms to describe the paper's scope. As a result, for each publication, we retrieved all articles published in the same year with at least one identical MeSH term[47]. We defined hit papers as being in the top 5% of citations received among all these retrieved publications. This method defines fields on the paper level instead of journal (set) level, which differs substantially from the Web of Science subject categories used in the main text. Performing this analysis, we arrived at the same conclusions, finding that our results are robust to this alternative field definition (Supplementary Figure 18).

Robustness for *ex post* funding status

In this section, we tested if the results are similar after controlling for *ex post* funding status. Due to the strong selection bias, we proceeded descriptively by comparing near misses ($N = 360$) with narrow wins ($N = 292$) conditional on securing a grant within 5 years after the treatment. We find that *ex post*, these near misses again outperformed these narrow wins, showing a strong support for the robustness of our results against this variation (Supplementary Figure 19).

Matching strategy and additional results in the RD regression

In this section, we tested whether the results are robust to matching strategy and some additional controls. First, to ensure publication time and PI past grant score do not affect our conclusions, we first controlled for publication year by adding publication year fixed effects. Publication year fixed effect allows us to estimate the effect of near miss on career impact only sampling articles

published within the same year. We uncovered the same results, and find our conclusions to be robust (Supplementary Figure 31 a, b, e, and f). Second, we also controlled for fixed effects categorizing PIs' prior grant experience. Specifically, we grouped all PIs into three different groups: PIs without prior grant applications, PIs whose most recent applications were discussed on the review panel (have scores), and PIs whose most recent applications were not discussed on the panel (no score, indicating that the application didn't pass the initial screening). By adding PI's past grant history fixed effects that only compare PIs within the same group, we find our results again to be robust (Supplementary Figure 31 c, d, g, and h). Together these results show that the performance advantage by the near misses cannot be simply explained by publication time and differences in PI's prior grant applications.

We further control for PI demographic features including the reputation of the scientific institution to which an applicant belongs (as measured by the number of successful NIH R01 awards from 1990 to 2005), gender and ethnicity, which may be sources for potential peer review bias[48,49]. We used a start-of-art ethnicity detector based on the first and last name[50], and apply it to the PIs in our sample. We find our results robust (Supplementary Figure 17). Specifically, we find that a single near miss increased the probability to publish a hit paper by 6.4% (*p*-value = 0.02), citations by 8.04 (*p*-value < 0.01), hit papers by 4.30 (*p*-value = 0.05) in the subsequent 10-year window conditional on active in the NIH system. The results hold for the conservative removal as well.

Next, we calculate the effect of early-career setback on paper applied values using the RD estimation. The dependent variable here are: 1) whether a paper is a clinical trial publication as indicated in the PubMed database (direct contribution to clinical translation); 2) whether a paper has been cited by clinical trial publications (indirect contribution to clinical translation); and 3) whether a paper has the potential to be translational research. Specifically, the Approximate Potential to Translate (APT) score was used to identify early signatures of bench-to-bedside translation[51, 52]. The score is estimated using a simple machine learning method combing features such as MeSH terms, disease, therapies, chemical/drug, and citation rates. A paper is considered as translational research if the score is above 0.5. Due to the lack of precision associated with the IV estimation (Wald test of exogeneity, *p*-value > 0.1, suggesting no evidence of correcting for endogeneity), we focus on OLS regression here. We find that an early-career near miss increases

the probability to publish a translational research by 4.7% (p-value = 0.05), to publish a clinical trial article by 0.9% (p-value = 0.12), to publish a paper that has been cited by clinical trial publications by 2.8% (p-value = 0.17).

Finally, we used the state-of-art matching strategy, i.e., Coarsened Exact Matching (CEM). The matching strategy future ensure the similarity between narrow wins and near misses *ex ante* (see Method section in the main text). We first show that the difference between matched near misses and narrow wins are indeed similar in terms of all observable dimensions (Supplementary Figure 20), with all measurements being statistical indistinguishable (p-value > 0.1). We then repeated all our analyses, finding within the matched samples, near misses again outperformed narrow wins in the subsequent 10 years after treatment, and the difference cannot be fully explained by the screening effect. We find the results are robust using CEM matching (Supplementary Figure 21).

On the screening mechanism

In this section, we conduct further analyses to test the underlying assumption of the screening mechanism. The screening mechanism hypothesizes that near misses who remained active in the next 10 years after setbacks are "better" *ex ante* than near misses who have been screened out. To test this hypothesis, we compared several pretreatment features between active and inactive near misses, finding near misses who have left the system are indeed weaker than those who manage to stay in (Supplementary Figure 29b). However, we conducted the same analysis for narrow wins, obtaining the same results (Supplementary Figure 29c), suggesting screening effect may occur, but if so, it runs in the same direction for both narrow wins and near misses. Indeed, we compared pre-treatment characteristics of near misses and narrow wins who remained active for the next ten years, finding a lack of difference between these two groups in any observable dimension *ex ante* (Supplementary Figure 29a). We also obtained the same results using 2SLS regression analysis with dependent variable being the probability to publish hit papers and average citations per paper *ex ante* (for both cases, p-value > 0.25). Together these results suggest that the screening effect might be modest, if exists at all.

Nevertheless, we further conducted the conservative estimation by removing "less able" narrow wins to create artificial upward adjustment for performance of those who remained. As shown in the main text, we find upon removal, the performance gap remained. In addition to the removal procedure outlined in the main text, we also tested other, more conservative methods by redefining the "less able" PIs based on the LHS against which we compare the two groups. That is, when comparing hit rate per paper, "less able" PIs are those publish the fewest hit papers with the most papers; when comparing the number of hit papers and the number of publications, "less able" PIs are those who publish the least hit papers or publications, respectively. Amid all these variations, we find the main result holds the same (Supplementary Figure 30).

Variance and outliers

Previous sections show that near misses show an average increase in citations, here we examine other measures to inform further the shift in distribution. First, near misses have lower chance to publish low quality research whose citation is below the average of all publications from the same field and same time (Supplementary Figure 23a, χ^2-test, p-value < 0.001). Second, we examine the coefficient of variation for citations of papers published by narrow wins and near misses in the next 10 years. The coefficient of variation (CV) is defined as $c_v = \frac{\sigma}{\mu}$, where σ is the standard deviation of the citation, and μ is the average citations. We see that near-miss applicants have a slightly higher coefficient of variation, but the difference is not significant (Modified signed-likelihood ratio test (SLRT) for equality of CVs p-value > 0.1, Supplementary Figure 23b). Finally, we further compared the median citations in order to eliminate the effect of outliers. Within the next ten years, the median citations of the near misses are substantially higher than that of narrow wins, and the difference between the two groups is statistically significant (two sample Mood's median test p-value < .001). This is also true for the case of the conservative removal procedure (two sample Mood's median test p-value = 0.017).

Different definitions of active PIs

In the main text, active PIs are measured through individual grant activities. Specifically, we trace all NIH grant activities for each PI, and define active PIs as those who applied for and/or received NIH grants at some point in the future, i.e. after the measurement time period. Correspondingly,

inactive PIs are those who neither applied for NIH grants nor had one beyond the measurement time period, identifying those who disappeared from the NIH system. Formally, let us denote the treatment time by t, and our measurement period by T_m ($T_m \leq 10$ years). A PI is considered as an active scientist in the NIH system she/he either applies for an NIH grant or receives one after $t + T_m$; she/he is defined as inactive otherwise. Note that this definition is a cumulative measurement. That is, once an NIH PI became inactive, he or she was never active again within the observation window covered by our data.

Finally, as an alternative robustness check, we modified the definition of active PIs by focusing on those who published at least one paper during a certain time window, i.e., PIs with at least one publication between t and $t + T_m$. We find the results are robust in terms of this alternative definition (Supplementary Figure 25). The near misses again outperformed the narrow wins in terms of hit rate per paper and average number of citations; the effect cannot alone be explained by the screening effect as the difference of these two groups is still significant after conducting the conservative removal.

Was it because narrow wins became worse?

In the main paper we documented a performance difference between the near-miss and narrow-win groups. But could it be simply because narrow wins became worse after the treatment? To test this hypothesis, we investigated another group of clear winners. We selected success applicants whose scores were further removed from the funding threshold (normalized priority score range from -20 to -10). We find that prior to treatment, across many metrics, there is a clear difference between the near misses and clear winners. For example, in the prior three years before treatment, clear-win applicants typically have a 1% higher hit rate per paper compared with near-miss applicants; the number of hit papers per person is 2.3 compared with 1.7 for near misses; the average citations for the clear winners is 13% higher than for near misses; and clear winners show more research experiences than near misses. This advantage is expected given our design, showing that the clear winners clearly outperformed the near misses prior to treatment. After the treatment, however, we find that near misses outperformed clear winners in terms of hit rate per paper if we only focus on active PIs. With the conservative removal, we find the two groups have very similar

performance 10 years after treatment in terms of different metrics (hit rate per paper, number of hit papers per capita, number of publications and average normalized citations in the 10 years after treatment, Supplementary Figure 24), indicating that near misses are now performing at a comparable level as the group that were demonstrably better than them prior to treatment. Given the fact that near misses are comparable with clear winners *ex post*, who showed significant advantages *ex ante*, the observed gap is unlikely solely due to the fact that narrow wins become weaker.

On this related point, we take a step further to check whether the results can also be accounted by the fact that narrow wins committed to their initial proposed "*sub-optimal*" ideas, while setbacks enabled near misses to further develop their ideas. Here we build on this idea to further interrogate our data. We have considered what kinds of analyses may help test this hypothesis and considered two predictions that may follow in line with this hypothesis:

- First, if narrow wins were initially committed to the "sub-optimal" ideas, it may suggest that their work in the more distant future would be significantly better than the research performed under this grant. We can therefore test this prediction by comparing their initial publications in the years after the grant with their later publications.
- Second, since the initial quality of these two groups are not different under the assumption of the RD (and reinforced by the tests on observables), after the grant that initially "locked in" a narrow win expires, later works by narrow wins may be more similar to those by near misses. This suggests that under the hypothesis we may observe a convergence of career impact between these two groups beyond the initial five years.

To test these predictions, we first compare publications by the narrow wins within 5 years after the grant approval with those published in the next 5 years (year 6--10). Papers published within the first 5 years were more likely to be the product of grant ideas, whereas later papers were more likely to be related to new ideas developed beyond the initial grant. After comparing their performance across these two periods, we found an at most modest and not statistically significant improvement for narrow wins in terms of probability to publish hit papers (χ^2-test, p-value = 0.14, Supplementary Figure 22A), or normalized citations (t-test, p-value = 0.13, Supplementary Figure 22B). Furthermore, there is no significant difference between publications from 1 to 5 years and 6

to 10 years for near misses, indicating their performance on these dimensions are stable (and consistent with results reported in Figure 2 of the manuscript). Finally, there is still a significant difference between narrow wins and near misses between year 6 to 10.

Together, these results are consistent with those reported in the paper, but run counter to the hypothesis that narrow wins were temporarily "locked in" to bad ideas whereas near misses were set free. Note that, there could be path dependencies that may potentially explain the long-run differences observed in Supplementary Figure 22, but nevertheless, these analyses are also consistent with other mechanisms tested in the original manuscript. For example, we did not find any significant change in impact associated with shifting future research directions (see "Combining hypotheses *A-D*" in Supplementary Note 4). Together these results suggest that being initially trapped by the grant topic is unlikely to drive the performance gap.

Comparing narrow wins and near misses using the percentile score

NIH uses percentile score of proposals in each NIH institute to determine the funding outcome. A percentile score of an application is calculated by comparing all applications by the same study section at its last three meetings[1], which could take place in different years. Prior studies[11] used the priority score instead of the percentile score to infer the cutoff, which is what we did in the main text. But, to ensure that the results still hold, here we repeated our analyses by using the percentile to define the cutoff in each NIH study section. Specifically, for each study section in each fiscal year, we define the cutoff score as the third worst percentile score of funded grant applications in each study section so that there are only two out-of-order funding applications beyond the cutoff. Because percentile score has a narrower range than prior score, we focus on applications around the cutoff (from -2 to 2). By comparing the near misses with narrow wins, we find near misses consistently show higher performance in the longer run (Supplementary Figure 26).

Supplementary Note 4: Potential generative processes

While the broad idea of a failure-driven boost may take many forms, several such mechanisms may be detectable from data in our context. There are several plausible hypotheses for such processes as we mentioned in the main text. Here, we demonstrate how we test these hypotheses.

Hypothesis A: Novelty

Did early-career setbacks prompt near misses to attempt more novel research, whereas narrow wins are bound to their original ideas? Existing studies have shown that high-novelty research tends to attract more citations in the long-run[53], especially when a small degree of highly novel combinations of prior knowledge are balanced with otherwise highly conventional combinations of prior knowledge[54]. To test this hypothesis, we analyzed: 1) whether near misses are more likely to publish high-novelty papers in the ten-year window following treatment compared to narrow wins; 2) whether near misses shift toward publishing high-novelty papers compared to their own publications prior to treatment, relative to any such shift among narrow wins. By calculating paper novelty and conventionality based on its combinations of prior work[54], we conducted a 2SLS regression with the dependent variable being the probability of publishing a high-novelty paper. In a separate regression, we study the tendency to produce high-conventionality papers in the next ten-year window. We find there is little evidence that the early-career setback may have any effect (Supplementary Figure 28, p-value = 0.291 for novelty; p-value = 0.51 for conventionality). Further, we tested if there is any effect of early-career setbacks on publishing articles that include the highly novel and highly conventional combinations of prior work that have been shown elsewhere to predict high impact, finding again insignificant results (p-value = 0.67).

Finally, we introduce a standard difference-in-difference (DID) specification, measuring the shift of near misses toward publishing novel papers, compared to what the near misses used to do and relative to narrow wins. More specifically, we run a regression with interaction terms to capture any differential shift among near misses:

$$P(high\ novelty\ publication) = \alpha\ near\_miss + \beta T_{post} + \gamma\ near\_miss * T_{post} + \epsilon, \quad (1)$$

where $near\_miss = 1$ if the PI is a near miss, 0 otherwise; $T_{post} = 1$ if after treatment, 0 otherwise. We find, comparing with narrow wins, near misses were no more likely to publish high-

novelty papers relative to their prior publications (coeff. = 0.02, standard error = 0.017, p-value = 0.302). We obtained similar results when focusing on the likelihood of publishing highly conventional papers or balanced high-novelty/high-conventionality papers (p-value > 0.1 in both cases). Overall, the effect of the early-career setback does not seem attribute to the hypothesis that near misses published more novel papers.

Hypothesis *B*: Collaboration effect

Did early-career setbacks lead junior scientists to seek out advantageous collaborations? Previous studies have shown that teams are often responsible for producing high-impact work[55,56], suggesting that a collaboration effect may be a plausible explanation for the performance gap. Having missed out on funding, the near misses may resort to collaborations with other authors; hence the increase in hit papers we observed may potentially reflect work performed with distinguished colleagues. To test this hypothesis, we first repeated our analyses by focusing only on lead-author publications for the two groups. Here we follow existing studies[57] and define lead-author publications as the first-author or last-author publications. We find that, by focusing on lead-author papers only, our conclusions remain the same (Supplementary Figure 13a-d). We further employed the conservative removal procedure, finding significant differences between the near misses and narrow wins (Supplementary Figure 13ef). Moreover, to rule out the possibility that near misses have to work in the lab of established colleagues' after missing the funding, we also focused on last-author publications only[57], uncovering the same results (Supplementary Figure 27).

We also tested whether there exists any effect of near miss on team size, the number of different affiliations, as well as the probability to publish first-author/middle-author/last-author articles in the next 10 years after treatment, finding insignificant results for all cases (Supplementary Figure 28, p-value > 0.1). Finally, we tested if near misses teamed up with higher-impact collaborators by calculating the highest h-index among collaborators for each publication. We find no support for the hypothesis that near misses worked with higher impact collaborators in the 10 years after treatment (Supplementary Figure 28, p-value > 0.1). Together, these results indicate that the observed effect is unlikely to be attributable to collaborations.

Hypothesis *C*: Research direction shifts

Near misses may shift their research directions while narrow wins continue in the direction that they initially proposed. Were near misses more likely to change their research focus to a different field, or perhaps a hot one that tends to garner higher impact? To test whether there is any research direction shift for near misses within 10 years after treatment, we estimated the effect of early-career setbacks on research directions. To quantify the research direction of a certain publication, we measured keywords overlap between each article published after "treatment" and the set of articles published *ex ante*. Here, we consider MeSH Headings from the PubMed dataset as keywords, and measure their similarities using the Jaccard index. Comparing publications before and after the treatment, we find little evidence that early-career setbacks affected the research direction, which is consistent with prior studies[33] (p-value > 0.7, Supplementary Figure 28).

To test if the near misses may be more likely to publish on hot topics, we estimated the effect of early-career setbacks on the probability to publish an article on hot topics. We quantify hot-topic papers as papers with the most frequently occurring MeSH terms (top 5%) across all papers in the same year[58]. If a certain fraction of a paper's MeSH Headings belong among those that most frequently occurred, the paper may be considered as a hot-topic paper. We tried several variations based on specific fractions and ran a 2SLS regression for each case, where the dependent variable is the probability of publishing a paper on such hot topics.

First, we define a hot-topic paper as a paper where at least one of its MeSH terms belongs to the most frequently occurring MeSH terms. Under this definition, we find no significant effect of early-career setbacks on the likelihood to publish hot-topic papers in the future (coeff.=-0.0009, p-value=0.37). Second, we define a hot-topic paper as one where the vast majority (over 90%) of its MeSH terms all belong to the most frequently occurring MeSH terms. We find again no significant effect (coeff.=2%, p-value=0.12, Supplementary Figure 28). We did observe some suggestive evidence when we define a hot-topic paper as half of its MeSH terms (50%) belonging to the most frequently occurring category (coeff.=4.2%, p-value=0.067), but the effect weakened following the conservative removal procedure (coeff.=3.9%, p-value=0.101). Note that, near misses and narrow wins have the same probability to publish papers with hot topics *ex-ante* (p-value > 0.1). Finally, we repeated the 2SLS regression on publishing hit papers when controlling

for hot topic papers, finding that, irrespective to the varied definitions, the result remains significant (p-value = 0.018 for the conservative removal), indicating that changing research direction alone cannot account for the main effect.

Hypothesis *D*: Changing institutions

Near misses may change research institutions with increased frequency following early-career setbacks, and such moves might expose these scientists to new sets of ideas or new collaborators. To test this hypothesis, we trace the physical mobility of each PI through their affiliations recorded in R01 applications, and calculate the probability of changing institutions in the ten-year time window. We find that near misses had a 40% chance to change their initial institution, whereas narrow wins had a 42% chance to move in the next ten-year window (p-value = 0.515). 2SLS regression yielded similar results (p-value = 0.235), suggesting that physical movements are unlikely to be the source of the observed performance gap.

Combining hypotheses *A - D*

To test if there is combining all above hypothesis explain our main findings, we controlled all mentioned processes and ran a 2SLS regression, as follows:

$$\textit{1}^{st}\textit{ stage: } F_j = \alpha_0 + \alpha_1 s_j + \alpha_2 s_j^2 + \cdots + \alpha_p s_j^p + \pi_i z_j + \theta X_{i,pre} + \delta X_{i,attr} + \mu_t + \eta_n + \eta_j, \quad (2)$$

$$\textit{2}^{nd}\textit{ stage: } y_{it} = \beta_0 + \beta_1 s_j + \beta_2 s_j^2 + \cdots + \beta_p s_j^p + \lambda \hat{F}_j + \gamma X_{i,pre} + \delta' X_{i,attr} + \mu_t' + \eta_n' + \epsilon_i, \quad (3)$$

where $X_{i,attr}$ is the above mentioned hypothesis, i.e., novelty, team size, number of different affiliations, hot topic, author order, MeSH Heading overlapping with prior publications, etc. Controlling all these parameters, we find our main effects remained, suggesting additional processes may be at work (coef. = 7.1% p-value = 0.012 for active PIs; coef. = 6.6% p-value = 0.021 for the conservative removal).

Supplementary Figures

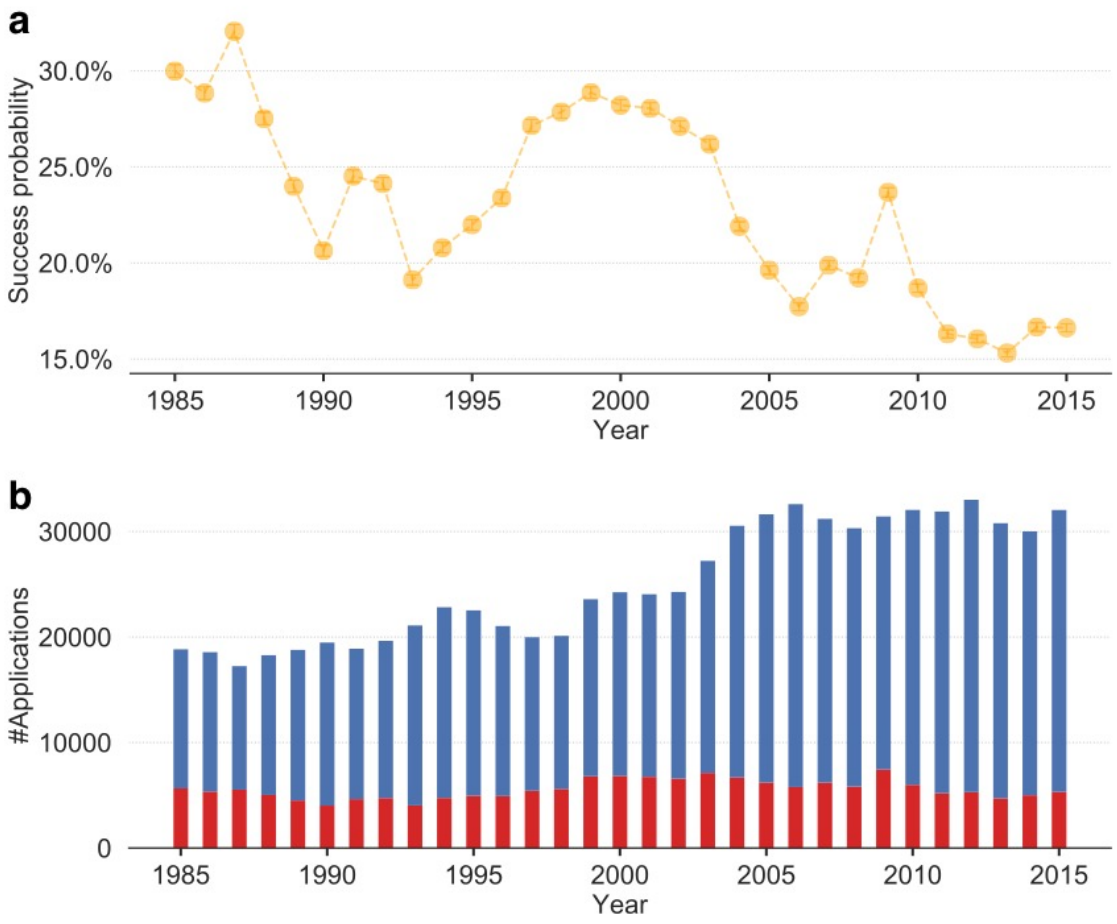

Supplementary Figure 1: NIH funding landscape. **(a)** R01 success probability as a function of time. **(b)** number of successful (red) and all R01 competing applications (blue) as a function of time.

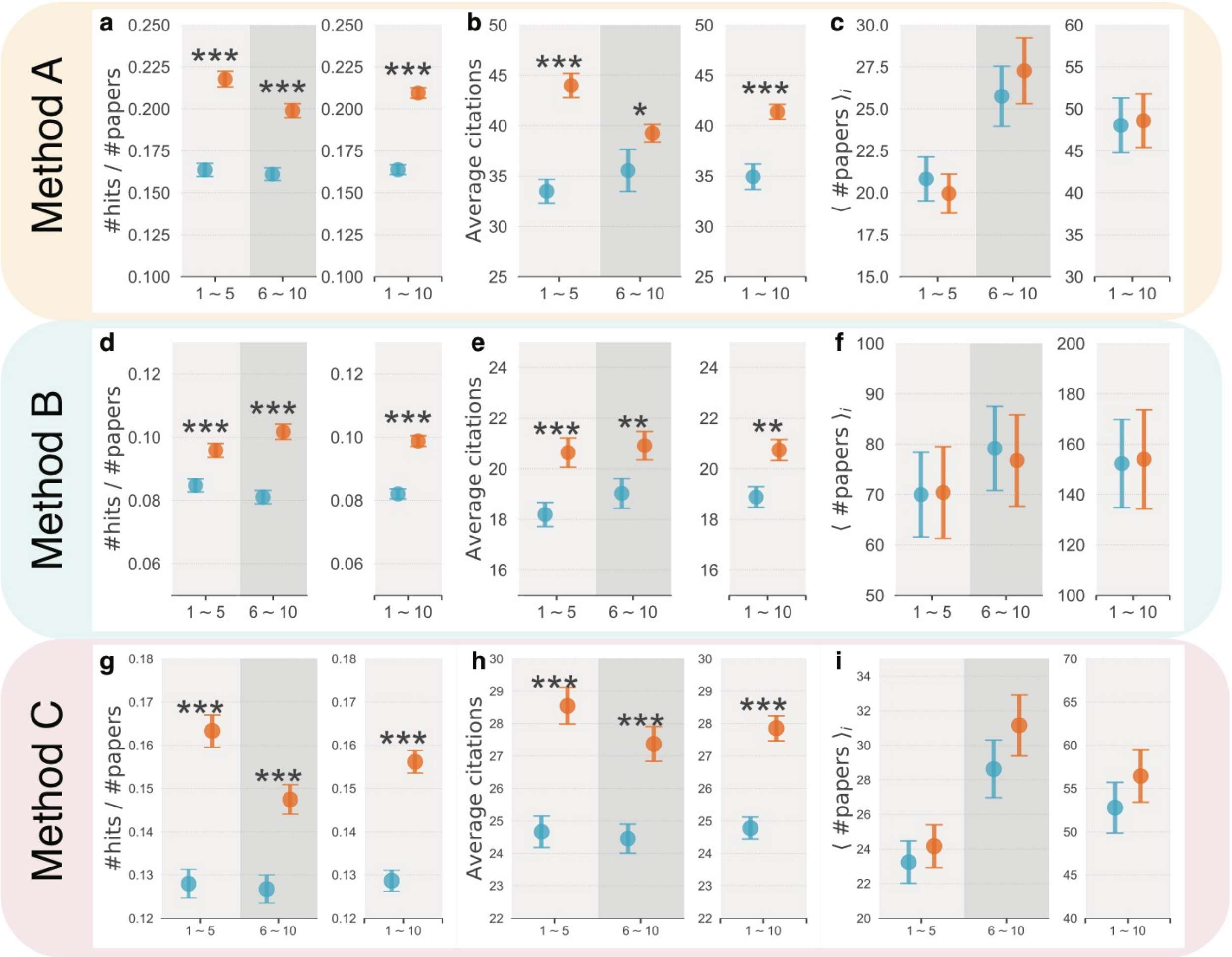

Supplementary Figure 2: Comparing narrow wins and near misses using different name disambiguation methods. The comparison of hit rate per paper (**a**), average citations within 5 years of publication (**b**), and (**c**) average number of papers between narrow wins and near misses. Here, if two papers from the initial pool are considered from the same author if they cite each other. (**d - f**) The same as (**a - c**), and two papers from the initial pool are considered from the same author if one of the following conditions is fulfilled: they cite each other, or share at least one common reference, or share at least one common coauthor name. **(g - i)** The same as **(a - c)** but for the name disambiguation method proposed by Caron and van Eck[9]. When calculating the average citations, we use the NIH applications from 1990 to 2000 in order to eliminate the boundary effect. *** $p < .001$, ** $p < .05$, * $p < .1$; Error bars represent the standard error of the mean.

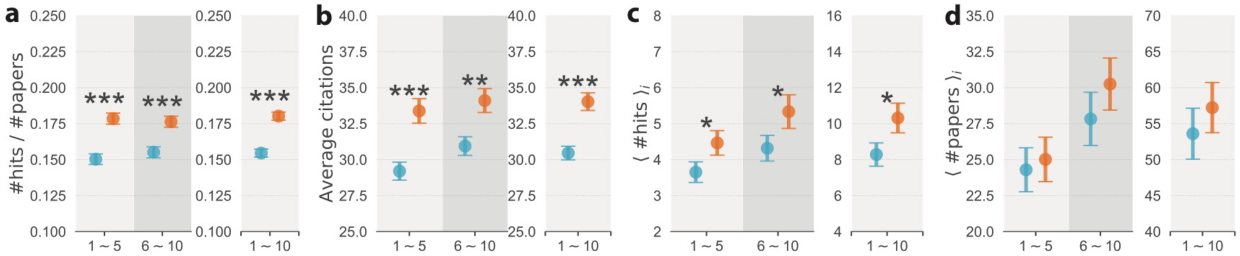

Supplementary Figure 3: Comparing near misses and narrow wins after removing PIs with common Asian surnames. (a) hit rate per paper; **(b)** average citations within 5 years of publications, here we focus on NIH grant applications from 1990 to 2000; **(c)** number of hit papers per person; **(d)** number of publications per person. *** p < .001, ** p < .05, * p < .1; Error bars represent the standard error of the mean.

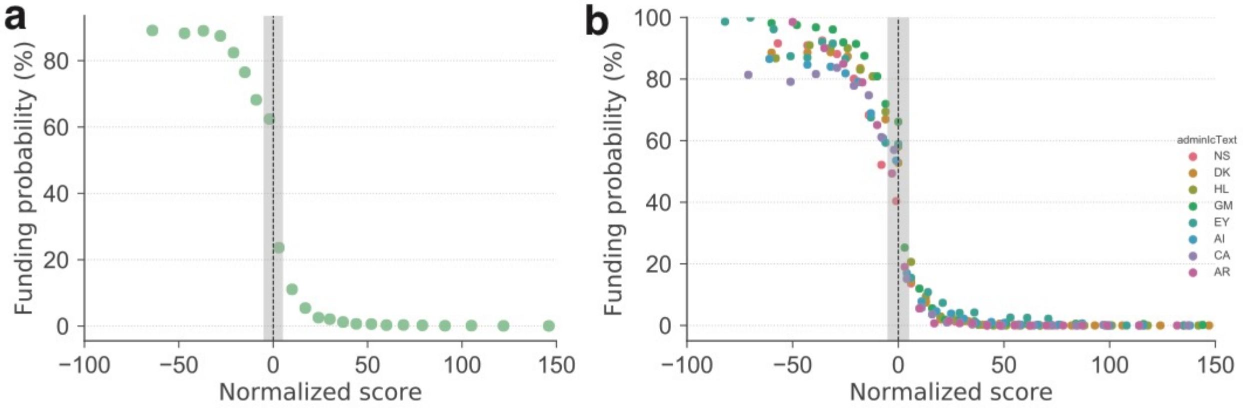

Supplementary Figure 4: Relationship between funding success probability as a function of score. (a) For all NIH grant applications considered in our study, the funding probability as a function of normalized score; **(b)** For major NIH institutes, which includes more than 75% of applications, the funding probability as a function of normalized score.

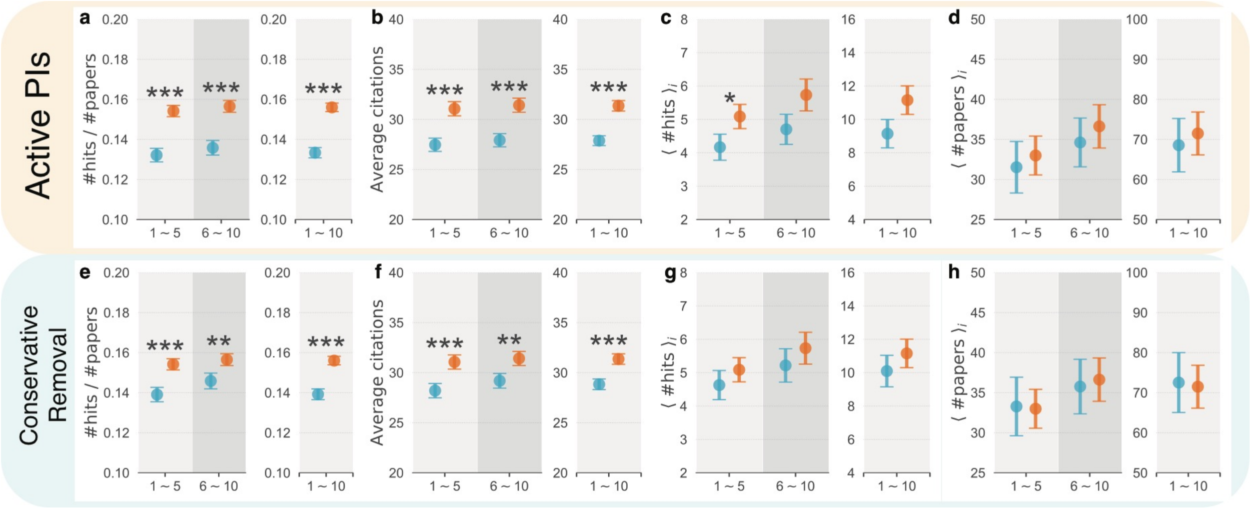

Supplementary Figure 5: Comparing near misses and narrow wins using cutoff as the second worst funded applications in each study section. **(a)** hit rate per paper; **(b)** average citations within 5 years of publications, here we focus on NIH grant applications from 1990 to 2000; **(c)** number of hit papers per person; **(d)** number of publications per person. **(e - h)** The same as **a - d** but using the conservative removal method. *** $p < .001$, ** $p < .05$, * $p < .1$; Error bars represent the standard error of the mean.

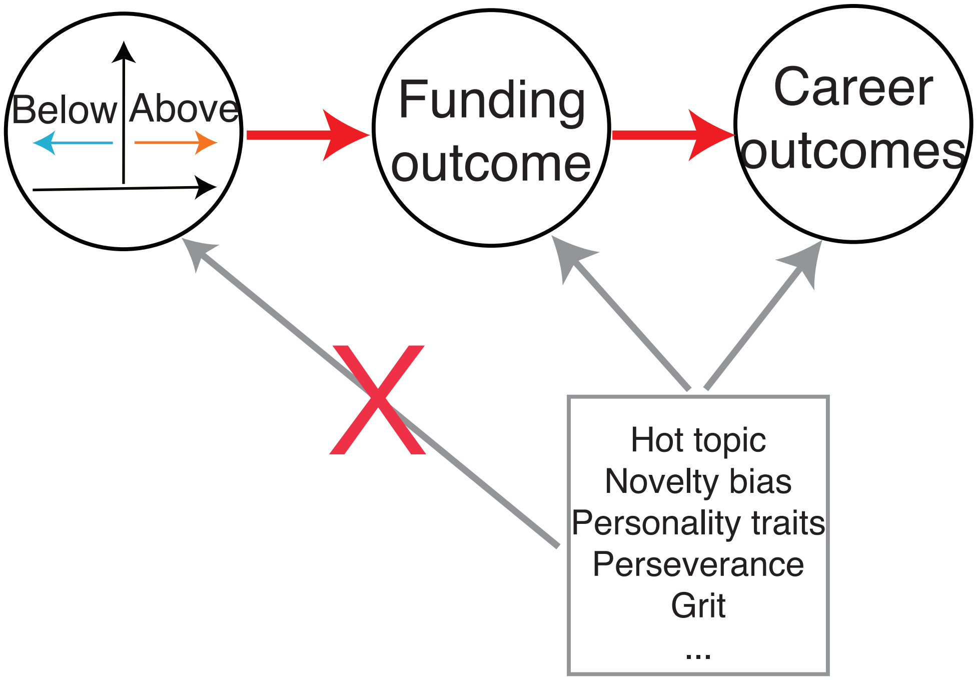

Supplementary Figure 6: An illustration of the fuzzy RD approach. In this figure, above or below the funding cutoff is treated as an instrumental variable (IV). The IV framework helps us disentangle unobserved factors by using variation created by the IV as an exogenous shock to one endogenous variable to estimate its causal effect on another variable. For example, there could be unobserved factors or individual characteristics that might influence both the funding and career outcomes (gray box and arrows), but these hidden variables differ smoothly with the score and are uncorrelated with the IV (gray arrow with a red cross). Whether or not one's score is above the threshold (the IV) only affects the funding outcome, but is uncorrelated with future career outcomes. Hence if the IV itself predicts future career outcomes, it would mean that the pathway indicated by red arrows must operate[59,60], allowing us to further establish a causal link between early-career near miss and longer-term scientific success.

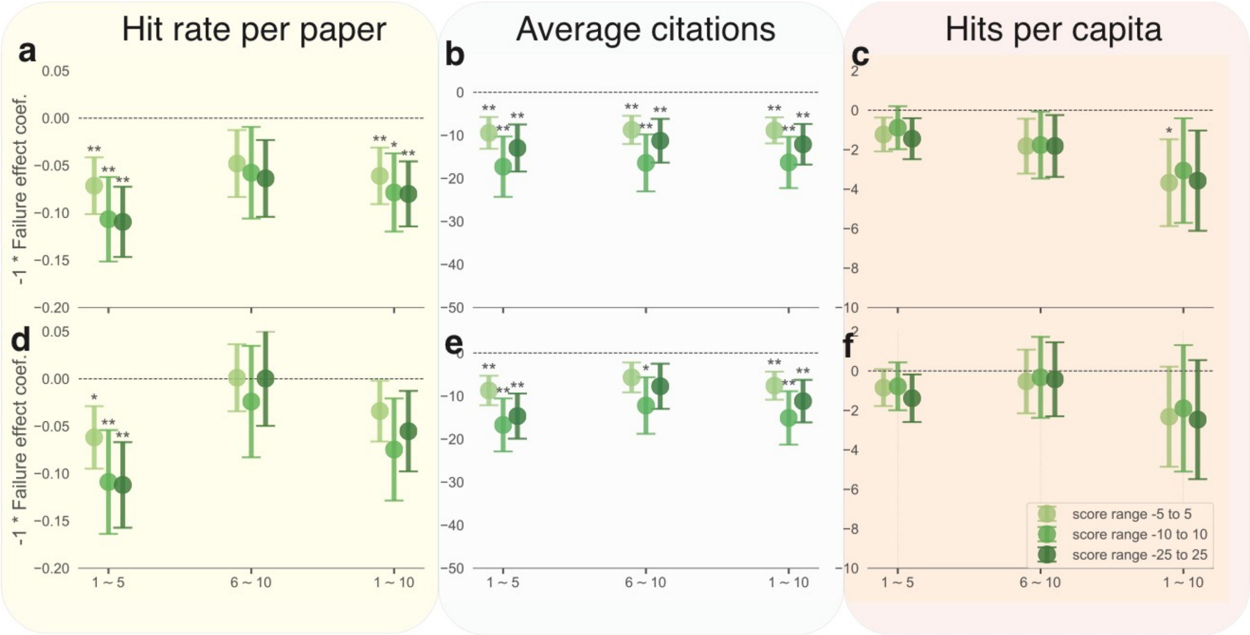

Supplementary Figure 7: The result from the fuzzy RDD estimation. (a) The effect of near miss on the probability to publish top 5% hit papers for applicants in the NIH system; **(b)** the effect of near miss on the average citations within 5 years after publication for applications in the NIH system; Here we use data from 1990 to 2000. **(c)** the effect of near miss on the number of top 5% hit papers published for applicants in the NIH system; **d - f** The same as **a - c** but for the conservative removal. Here, we use three different sample size, i.e., 5-score around the cutoff, 10-score from the cutoff, and 25-score from the cutoff. We add exclusive including NIH institution and time fixed effect, and PI prior performance (see Method section of the main text). Error bars represents the standard errors, and are clustered at individual level. *** p < .001, ** p < .05, *p < .1.

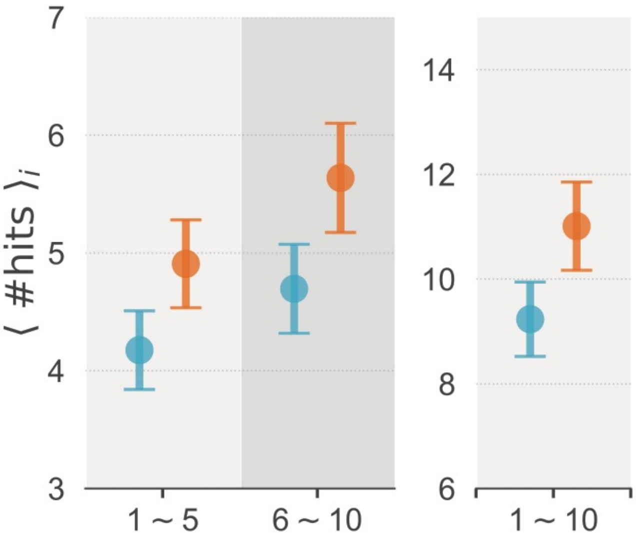

Supplementary Figure 8: Comparing hit papers per capita between near misses (orange) and narrow wins (blue). Hits per capita measure the number of hit papers per person. We uncovered the same direction of results as Figure 2 in the main text, albeit with a lesser significance level due to a reduced sample size (*p*-value = 0.107). Error bars represent the standard error of the mean.

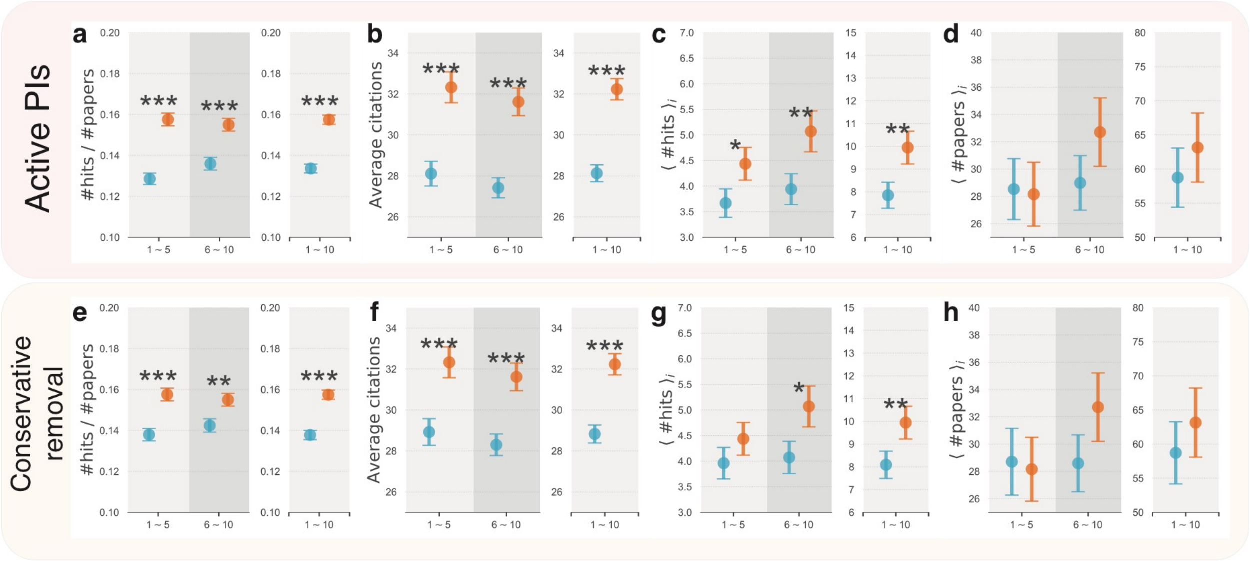

Supplementary Figure 9: Comparing near misses (orange) with narrow wins (blue) for those who applies for the first R01 grant at the time of treatment. a - d, The comparison of hit rate per paper, average citations within 5 years after publication (using data from 1990 to 2000), hits per capita and number of publications per capita for active PIs; **e - h,** The same as **a - d** but for the conservative removal. *** $p < .001$, ** $p < .05$, *$p < .1$; Error bar represents the standard error of the mean.

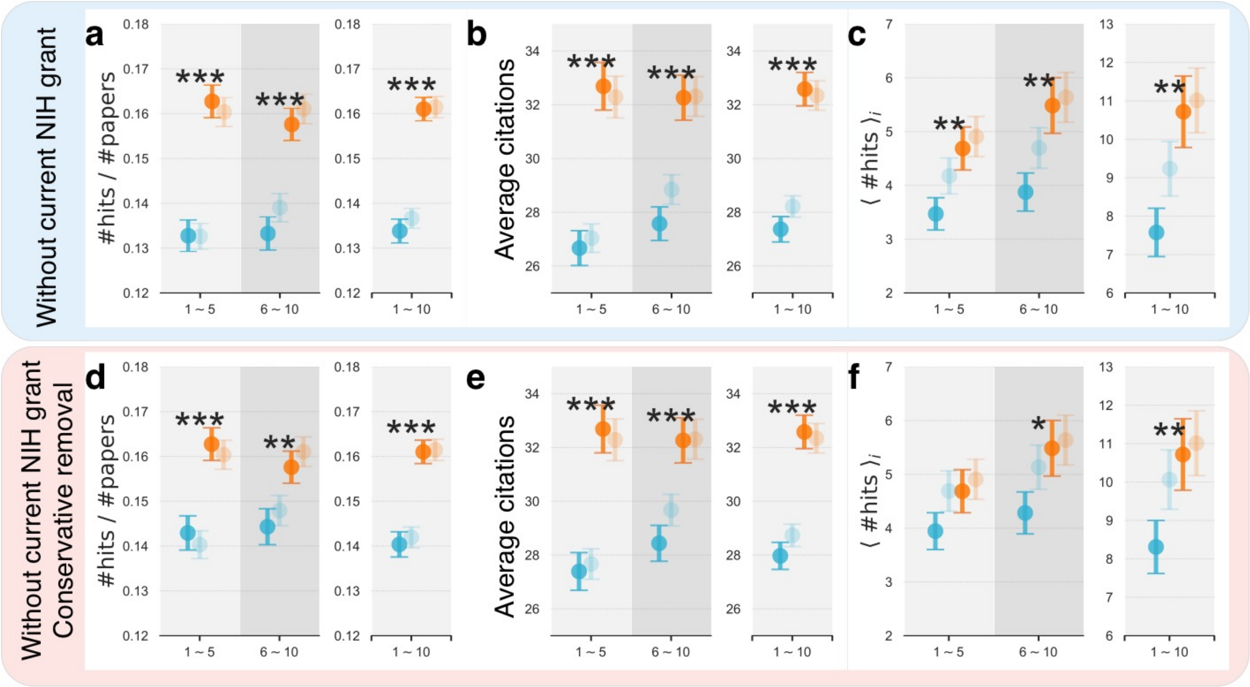

Supplementary Figure 10: The effect of a near miss magnifies when the stake gets higher. a - c, The subsequent performance by near misses and narrow wins, measured by the probability of producing a hit paper (**a**), average citations of papers attained within 5 years of publication; here we used data from 1990 to 2000 (**b**), and hit papers per capita (**c**). Shaded symbols represent the corresponding measurements reported in Fig. 2 of the main text. **d - f**, The same as **a - c** but for the conservative removal. Shaded symbols represent the corresponding measurements reported in Fig. 3 of the main text. *** $p < .001$, ** $p < .05$, *$p < .1$. Error bar represents the standard error of the mean.

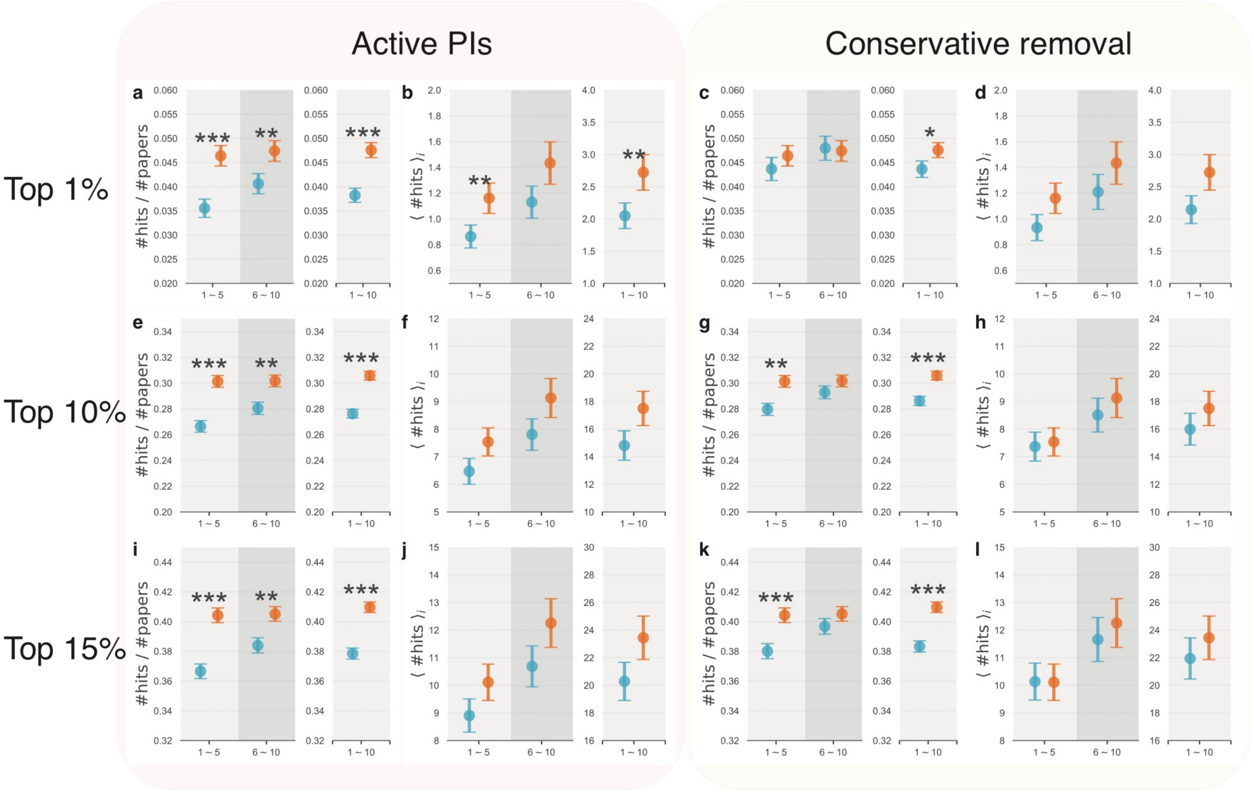

Supplementary Figure 11: Robustness check for various hit paper thresholds. Comparison between narrow wins and near misses using different thresholds to define hit papers. **a-d,** top 1% highly cited papers in the same field and same year; **e-h,** top 10% highly cited papers; **i-l,** top 15% highly cited papers. *** p < .001, ** p < .05, *p < .1; Error bar represents the standard error of the mean.

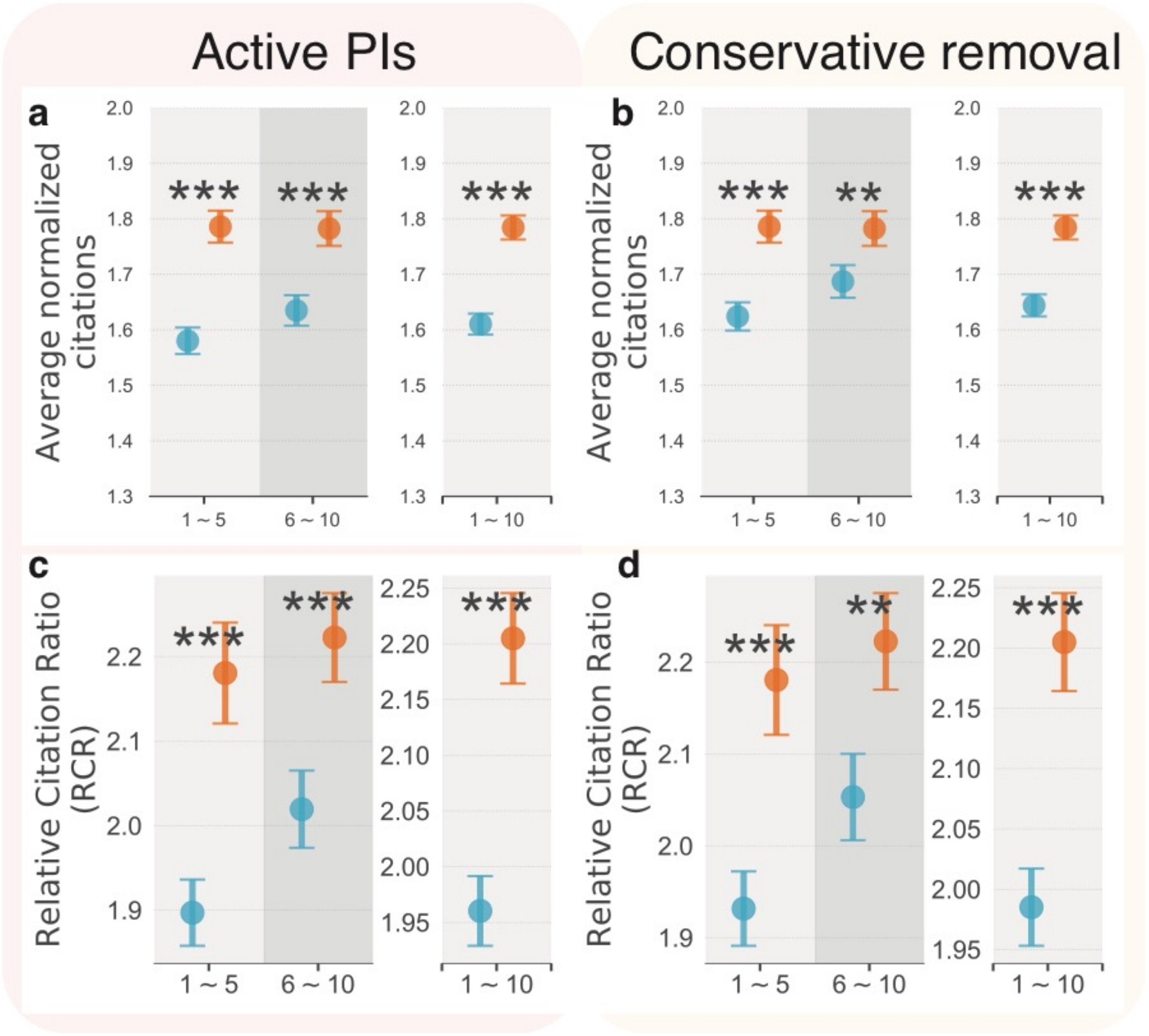

Supplementary Figure 12: Robustness check for the normalized citations with respect to field and time. Comparing normalized citations c_f between narrow wins and near misses for **a)** active PIs and **b)** conservative removal. Comparing the relative citation ratio (RCR) between narrow wins and near misses for **c**) active PIs and **d**) conservative removal. *** p < .001, ** p < .05, *p < .1; Error bar represents the standard error of the mean.

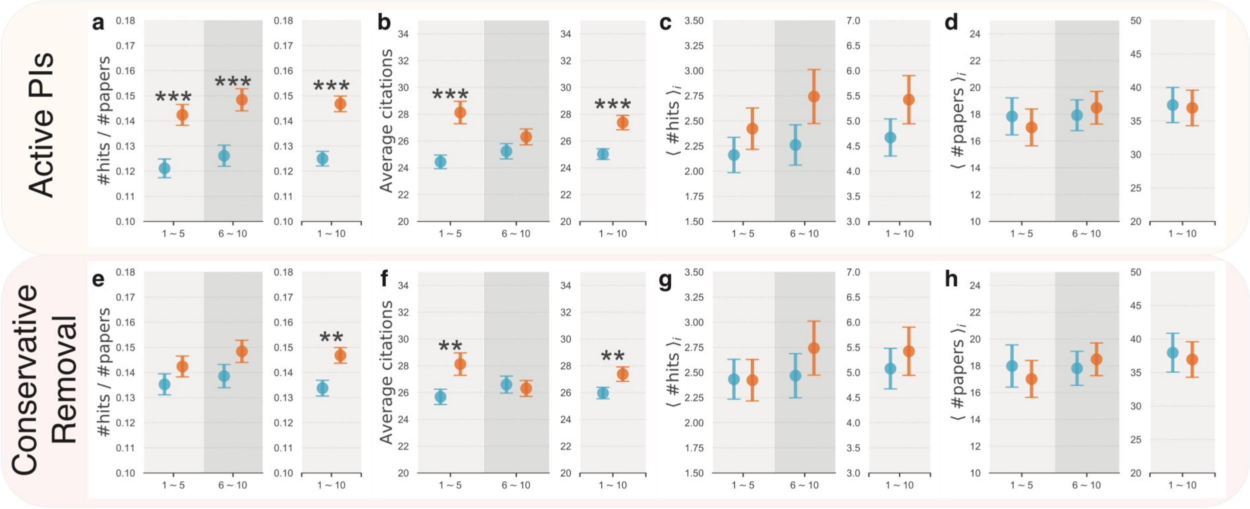

Supplementary Figure 13: Robustness check for lead-author publications. **a-d,** Comparing narrow wins with near misses in terms of hit rate per paper, average citations within 5 years after publication (using data from 1990 to 2000), hits per capita, and number of publications per person for active PIs; **e-h,** The same as **a-d** but we use the conservative removal method to account for the screening effect. *** p < .001, ** p < .05, *p < .1; Error bar represents the standard error of the mean.

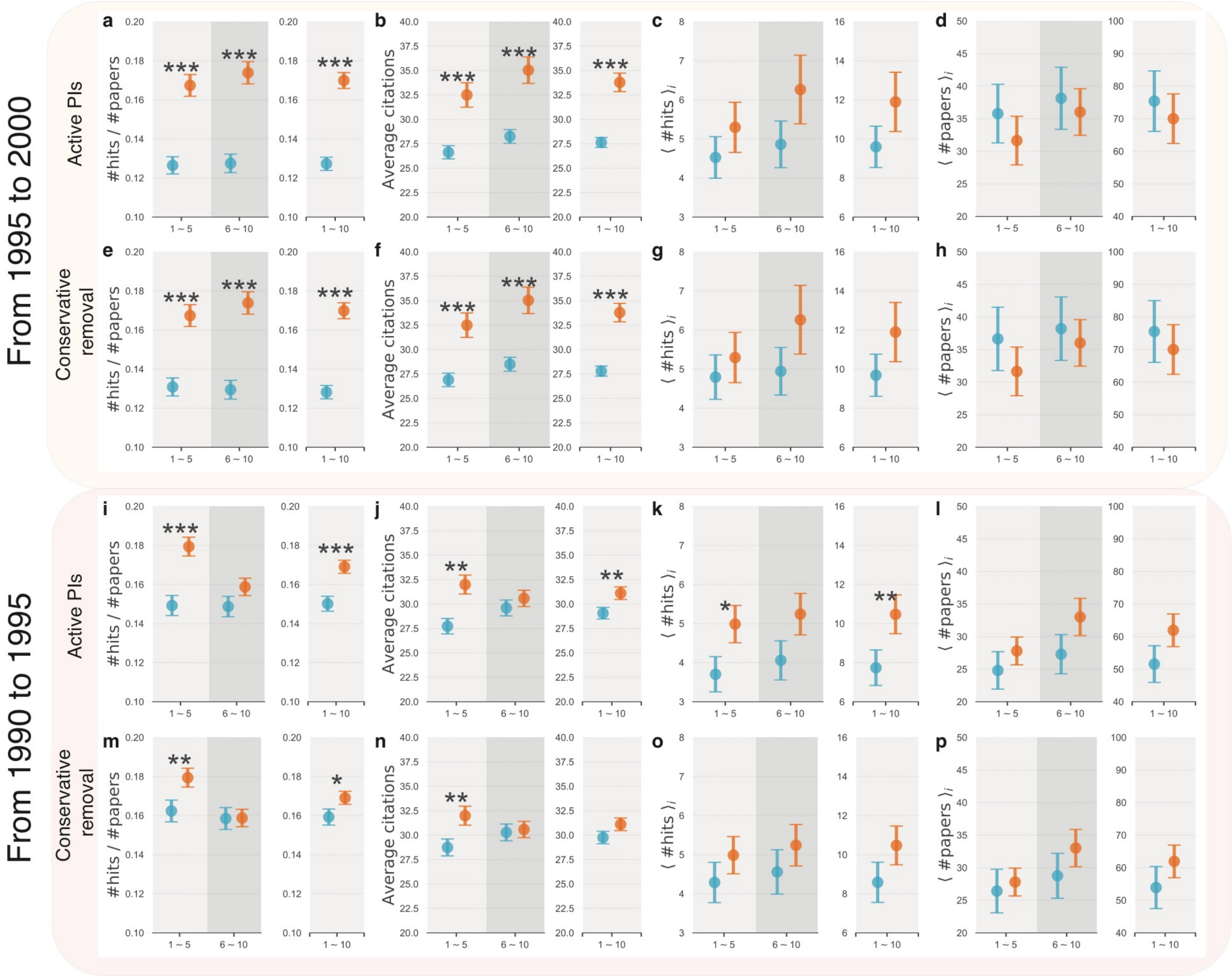

Supplementary Figure 14: Robustness check for different time windows. a-d, The comparison between near misses and narrow wins in terms of hit rate per paper, average citations per paper after five years of publication, hits per capita, number of publications per person for top 5% paper between 1995 to 2000 for active PIs; **e-h,** The same as **a-d** but for the conservative removal. **i-l,** The comparison between near misses and narrow wins in terms of hit rate per paper, average citations per paper after five years of publication, hits per capita, number of publications per person for top 5% paper between 1990 to 1995 for active PIs; **m-p,** The same as **i-l** but for the conservative removal. *** $p < .001$, ** $p < .05$, *$p < .1$; Error bar represents the standard error of the mean.

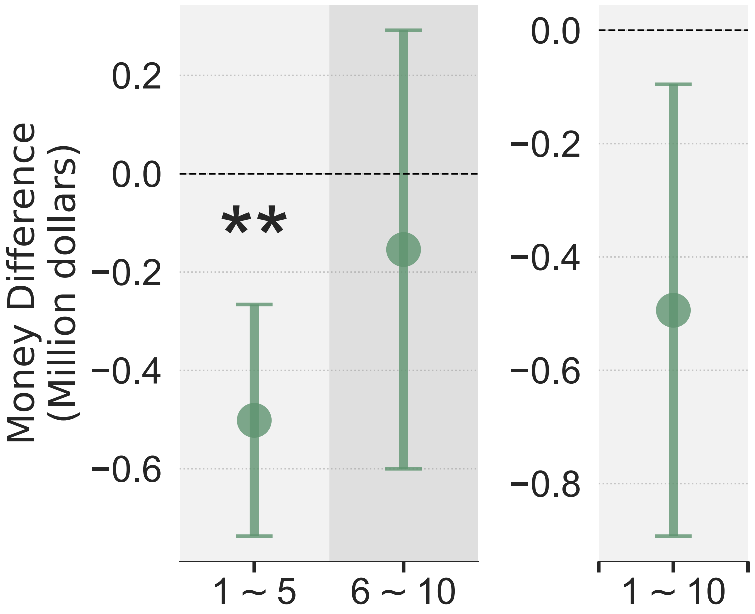

Supplementary Figure 15: Comparing the NSF money between near misses and narrow wins (near misses minus narrow wins) for two periods (1-5 years, and 6-10 years) using the Dimensions data. *** p < .001, ** p < .05, *p < .1 and NS for p > .1; Error bar represents the standard error of the mean.

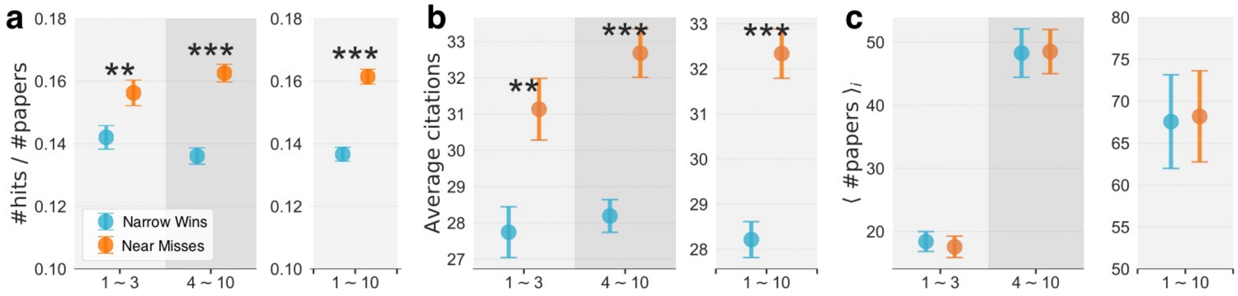

Supplementary Figure 16: Robustness check for publication lags. (**a**) Near misses outperformed narrow wins in terms of the probability of producing hit papers in the next 1-3 years, and 4-10 years. (**b**) The average citations within 5 years of publication. To ensure all papers have at least 5 years to collect citations, here we used data from 1990 to 2000 to avoid any boundary effect. (**c**) The average number of publications per person. *** $p < .001$, ** $p < .05$, * $p < .1$; Error bars represent the standard error of the mean.

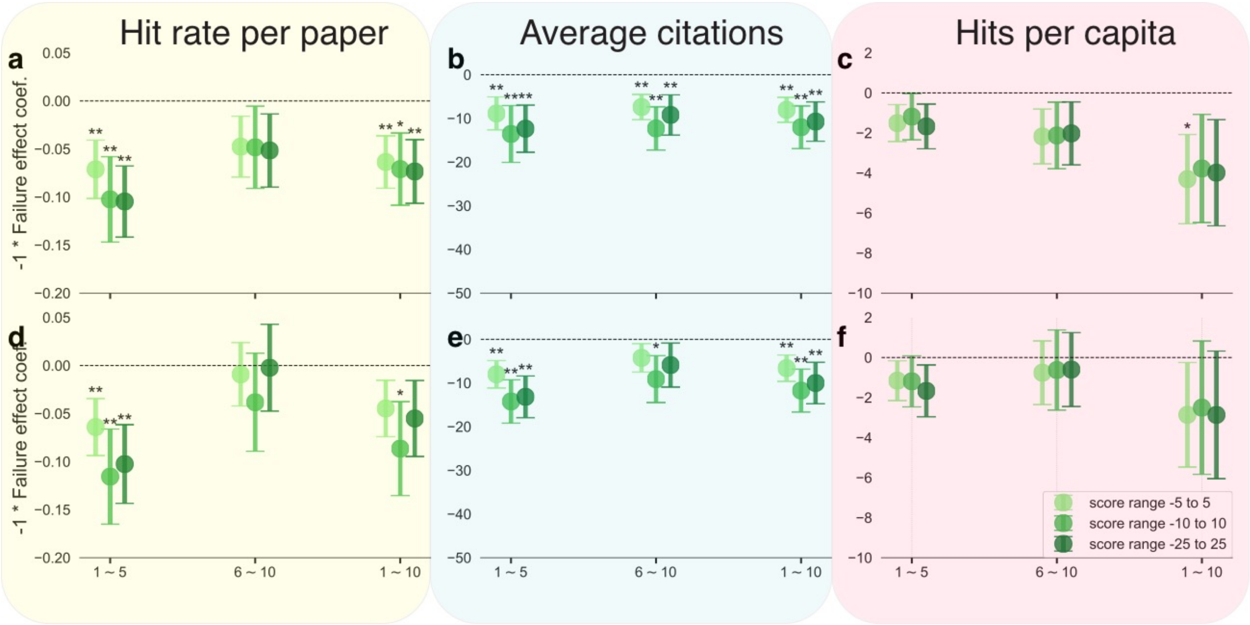

Supplementary Figure 17: The results from the fuzzy RD estimation after controlling demographic features and publication years. (**a**) The effect of near miss on the probability to publish top 5% hit papers for applicants in the NIH system; (**b**) the effect of near miss on the average citations within 5 years after publication for applications in the NIH system; Here we use data from 1990 to 2000. (**c**) The effect of near miss on the number of hit papers for applicants in the NIH system. Here we only control demographic features. (**d - f**) The same as **a - c** but for the conservative removal. Here, we use three different sample size, i.e., 5-score around the cutoff, 10-score from the cutoff, and 25-score from the cutoff. Error bars represent the standard errors, and are clustered at individual PI level. *** p < .001, ** p < .05, *p < .1.

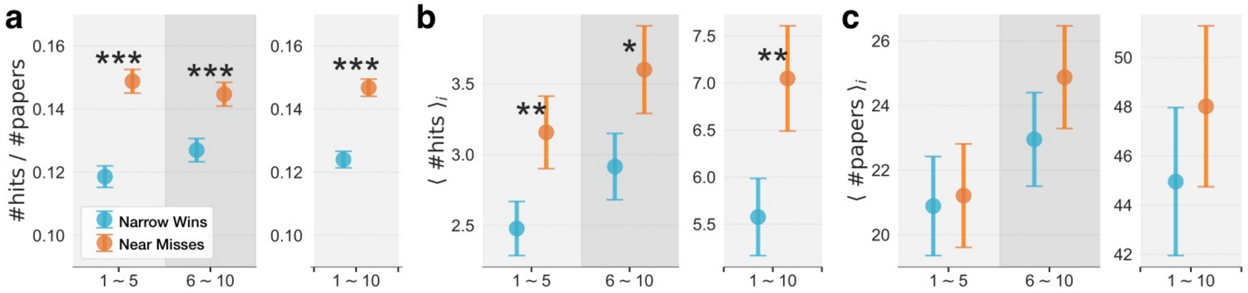

Supplementary Figure 18: Robustness checks using MeSH terms to define fields. (**a**) Near misses outperformed narrow wins in terms of the probability of producing hit papers in the next 1-5 years, 6-10 years, and 1-10 years. (**b**) Average number of hit papers. The near-miss applicants again outperformed their narrow-win counterparts. (**c**) Average number of publications. *** p < .001, ** p < .05, * p < .1; Error bars represent the standard error of the mean.

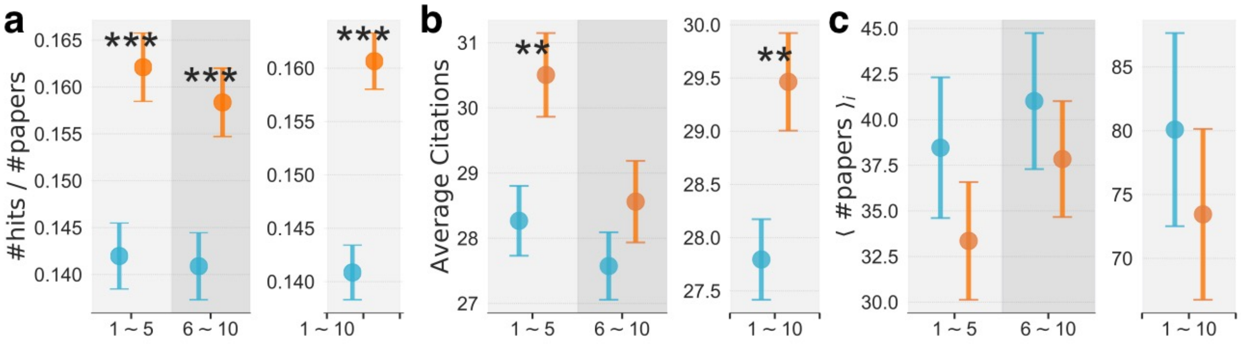

Supplementary Figure 19: Comparing near misses (orange) with narrow wins (blue) conditional on securing another successful grant within 5 years of treatment. (**a**) Near misses outperformed narrow wins in terms of the probability of producing hit papers in the next 1-5 years, and 6-10 years. (**b**) The average number citations within 5 years of publication. To ensure all papers have at least 5 years to collect citations, here we used data from 1990 to 2000 to avoid any boundary effect. (**c**) The average number of publications per person. *** p < .001, ** p < .05, * p < .1; Error bars represent the standard error of the mean.

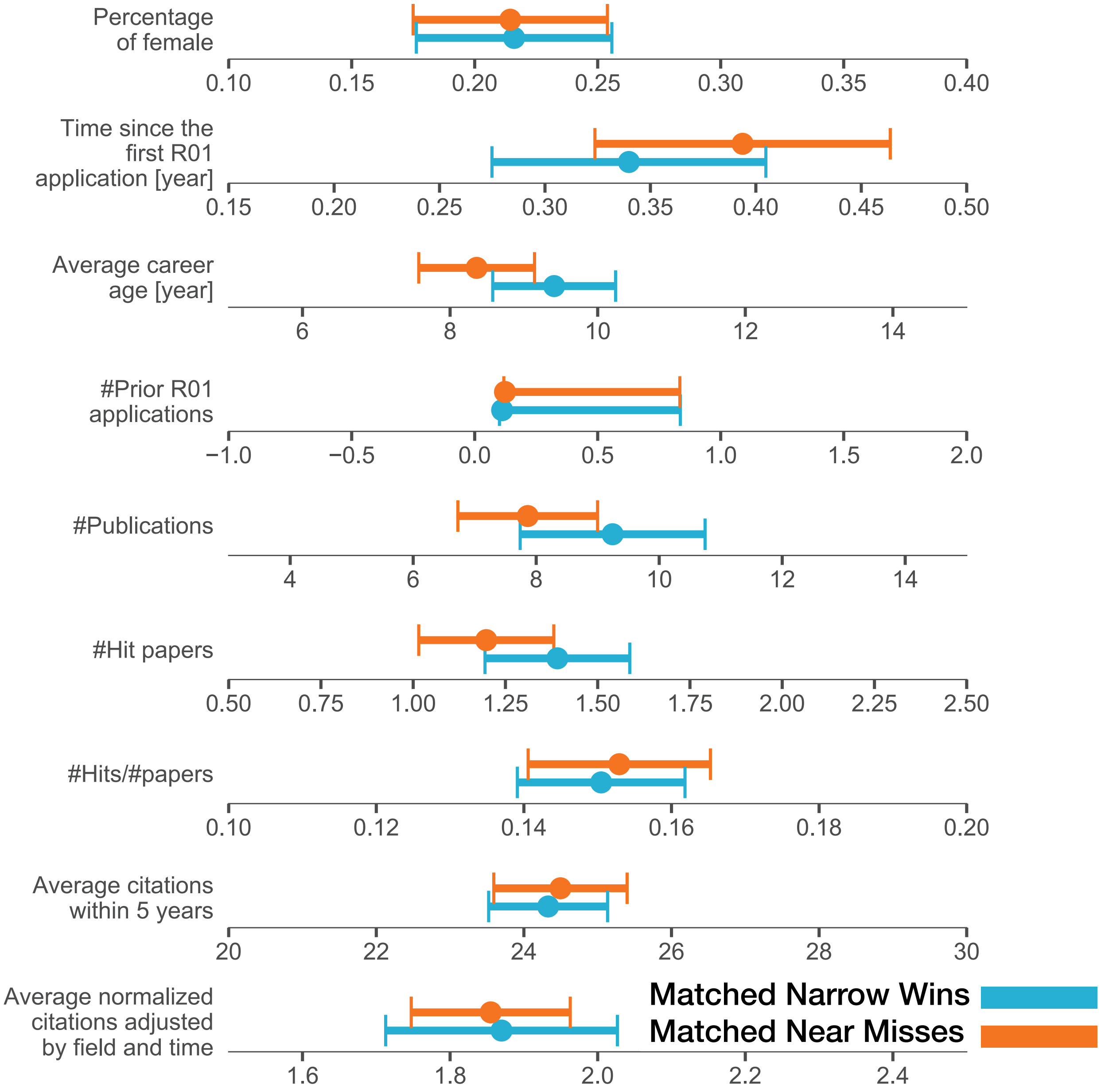

Supplementary Figure 20: Comparing near misses (orange) and narrow wins (blue) *ex ante* using CEM matching. Pre-treatment feature comparisons between the near-miss and narrow-win group. We compared various demographic and performance characteristics. The features are defined as follows (from top to bottom): 1) percentage of female applicants; 2) number of years since the first R01 application; 3) number of years since the first publication; 4) number of previous R01 applications; 5) number of publications prior to treatment; 6) number of prior papers that landed within the top 5% of citations within the same field and year; 7) probability of publishing a hit paper; 8) average citations papers received within 5 years of publication; and 9) citations normalized by field and time. We see no significant difference between the two groups across any of the ten dimensions we measured; Error bar represents the 95% confidence interval.

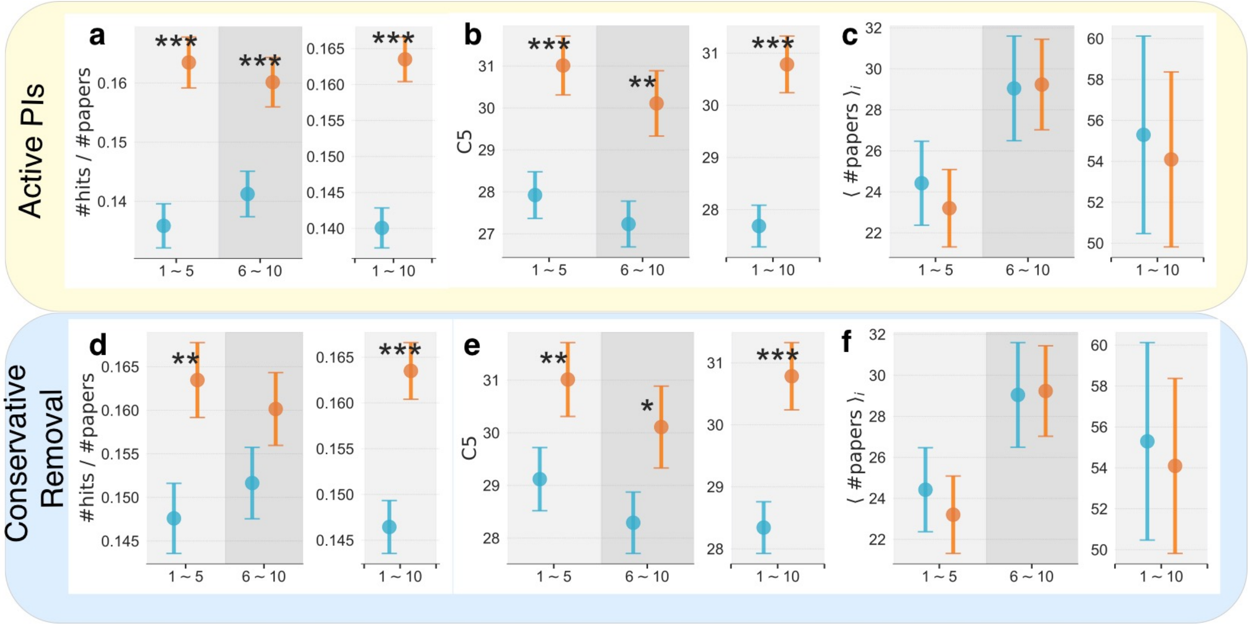

Supplementary Figure 21: Comparing near misses (orange) with narrow wins (blue) using the CEM matching. (**a**) Near misses outperformed narrow wins in terms of the probability of producing hit papers. (**b**) The average number citations within 5 years of publication. To ensure all papers have at least 5 years to collect citations, here we used data from 1990 to 2000 to avoid any boundary effect. (**c**) The average number of publications per person. (**d - f**) The same as **a – c** but for the conservative removal. *** p < .001, ** p < .05, * p < .1; Error bars represent the standard error of the mean.

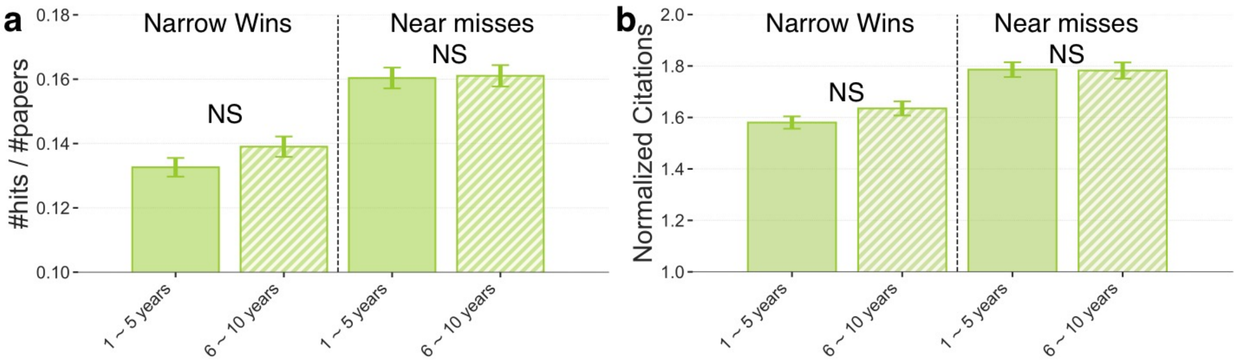

Supplementary Figure 22: Comparing future career outcome between publications within 5 years and from 6 to 10 years after the treatment, for both near misses and narrow wins. (a) Comparing hit rate probability, and we find there is no statistical significant difference between publications from the first 5 years and those from the second 5 years. **(b)** The same as **a** but for the average normalized citations per paper. We find there is no significant improvement for narrow wins. *** p < .001, ** p < .05, * p < .1; Error bars represent the standard error of the mean.

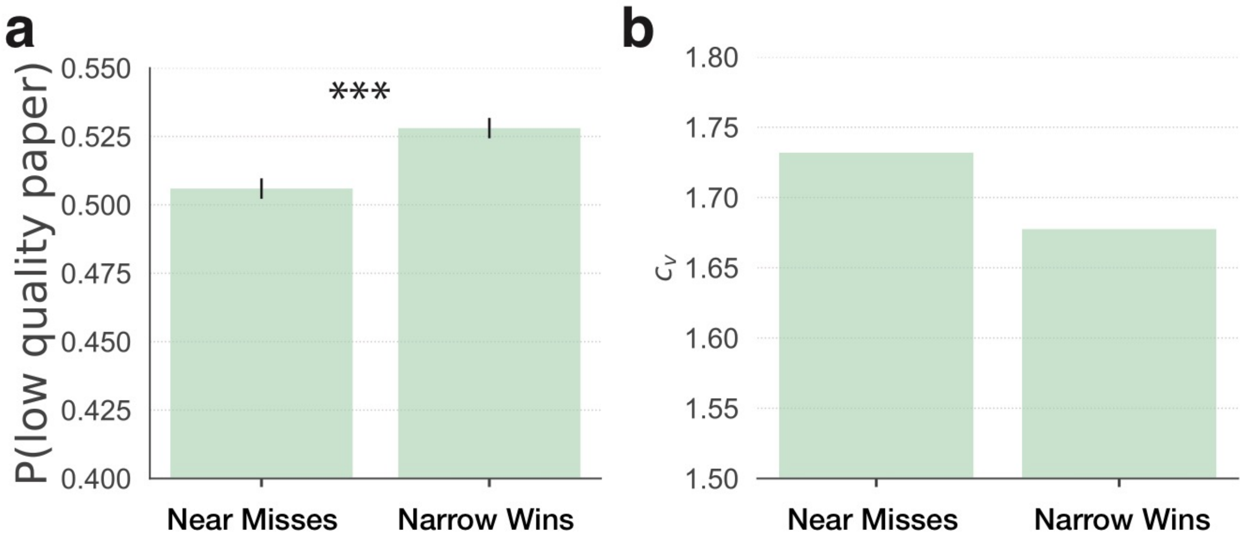

Supplementary Figure 23: Comparing citation uncertainty between near misses and narrow wins. (**a**) Probability to publish low quality papers, with citations lower than average citations of papers published in the sae field and time; Error bar represents the standard error of the mean. **(b)** Coefficient of variance of c_f for both groups. *** p < .001, ** p < .05, *p < .1; Error bars represent the standard error of the mean.

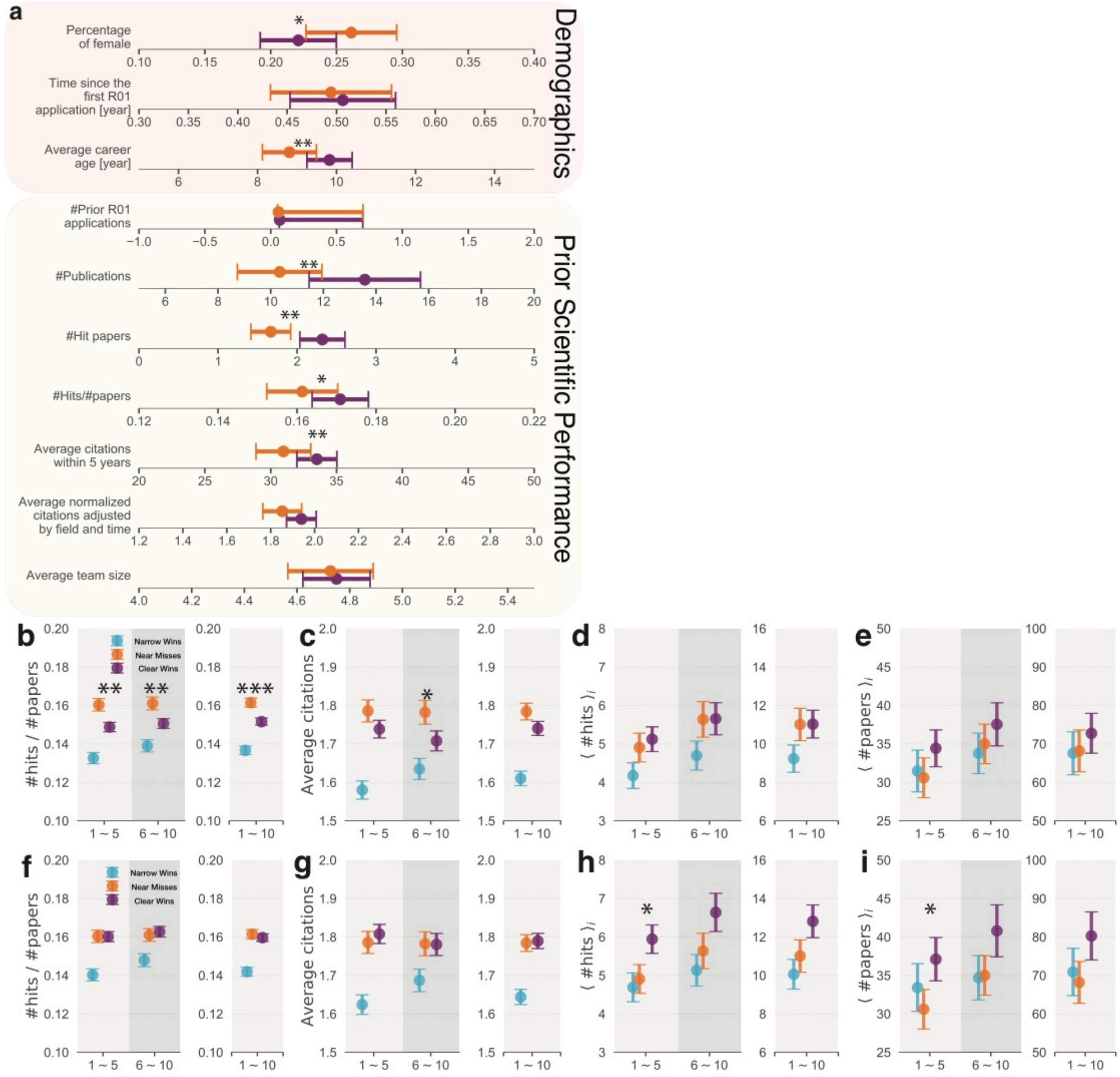

Supplementary Figure 24: Comparing near misses with clear winners in terms of various measurements, with the basis of narrow wins. (a) Comparing features of near misses with clear winners prior to treatment; detailed definitions can be found at Fig. 1 of the main text. Error bar represents the 95% confidence interval; **b-e,** comparing hit rate per paper (**b**), average normalized citations (**c**), number of hit papers per capita (**d**), number of papers (**e**); **f-i,** The same as **b-e**, but for the conservative removal. *** p < .001, ** p < .05, *p < .1; Error bar represents the standard error of the mean.

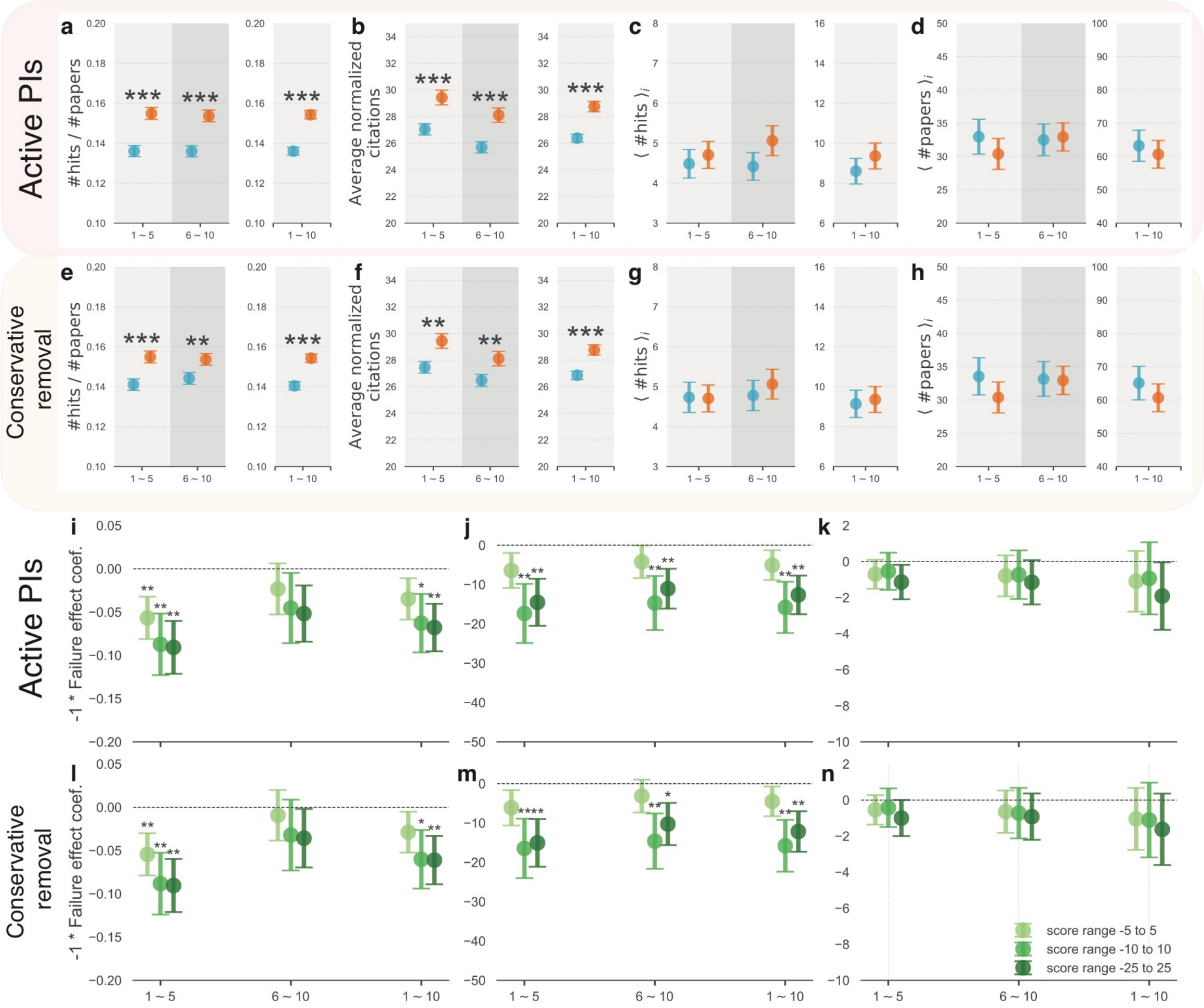

Supplementary Figure 25: Comparing near misses with narrow wins, here the active PI is defined as those who published at least one paper during certain time windows. a-d, The comparison between near misses and narrow wins in terms of hit rate per paper, average citations within 5 years after publication (using data from 1990 to 2000), hits per capita and papers per capita by focusing only on active PIs; **e-h,** The same as **a-d** but for conservative removal; Error bar represents the standard error of the mean. **i-k,** fuzzy RDD estimation of active PIs; **l-n,** fuzzy RD estimation of PIs after the conservative removal. In the regression estimation, error bars are standard errors clustered at individual level. *** p < .001, ** p < .05, *p < .1.

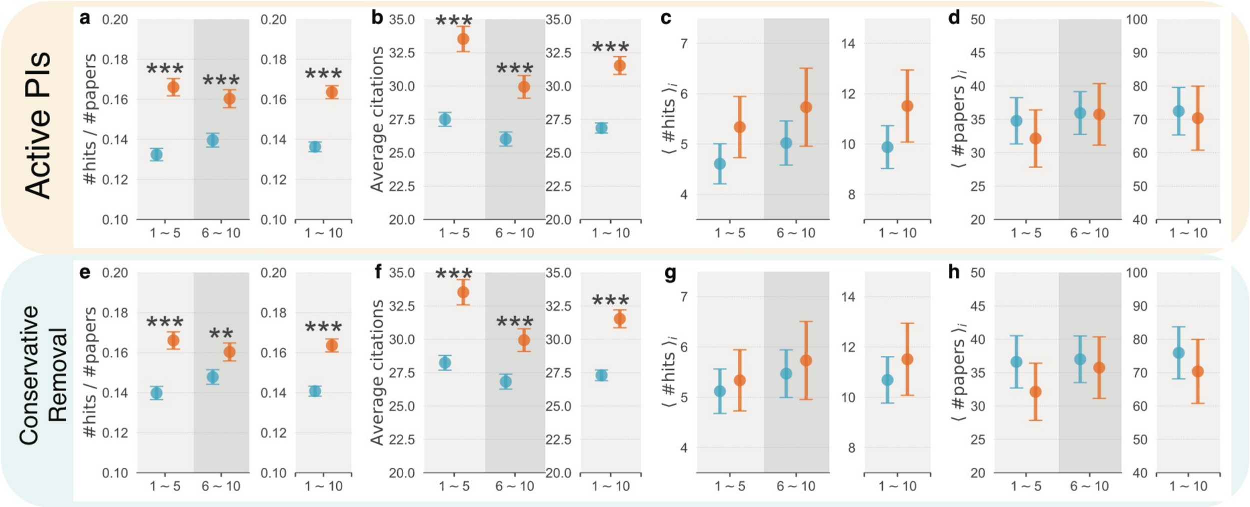

Supplementary Figure 26: Comparing near misses with narrow wins using cutoff defined by the percentile score. a-d, Comparing narrow wins with near misses in terms of hit rate per paper, average citations within 5 years after publication (using data from 1990 to 2000), hits per capita, and number of publications per person for active PIs; **e-h,** The same as **a-d** but we use the conservative removal method to account for the screening effect. *** p < .001, ** p < .05, *p < .1; Error bar represents the standard error of the mean.

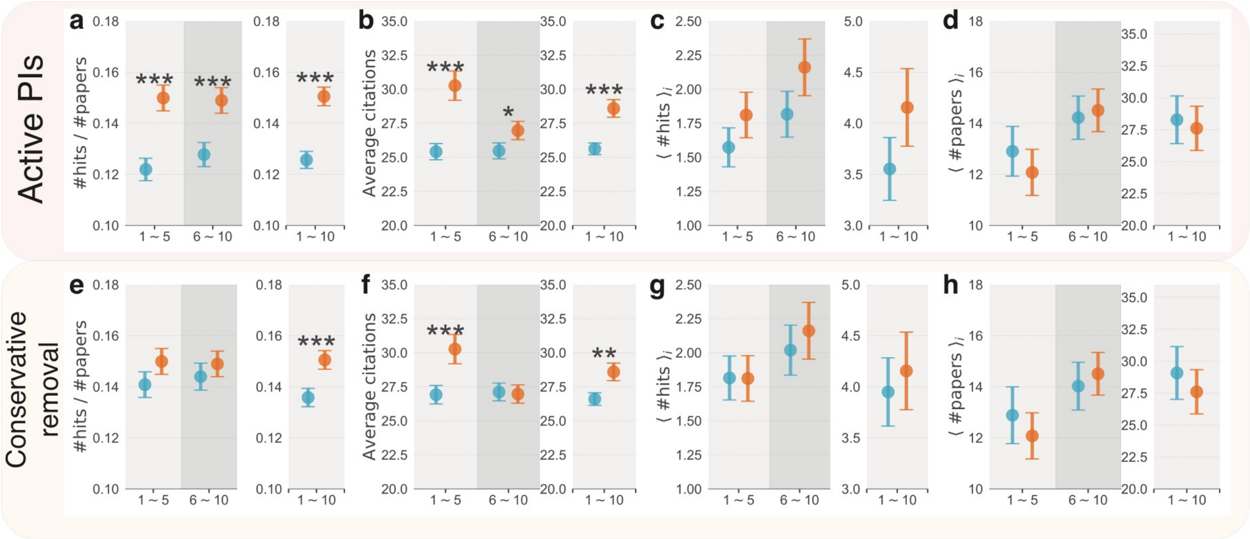

Supplementary Figure 27: Comparing near misses with narrow wins using last-author publications only. **a-d,** Comparing narrow wins with near misses in terms of hit rate per paper, average citations within 5 years after publication (using data from 1990 to 2000), hits per capita, and number of publications per person for active PIs; **e-h,** The same as **a-d** but we use the conservative removal method to account for the screening effect. *** p < .001, ** p < .05, *p < .1; Error bar represents the standard error of the mean.

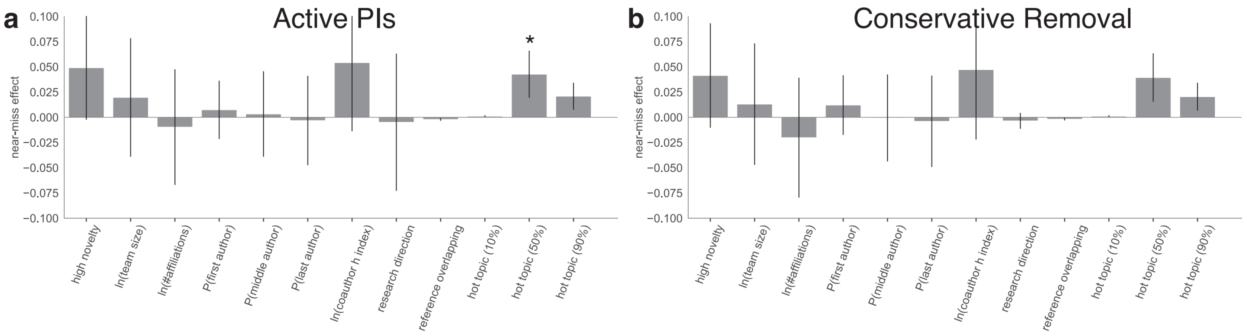

Supplementary Figure 28: Possible generative mechanisms of why near misses become better than narrow wins. **(a)** We present 2SLS regression estimates, studying the effect of early-career setbacks on ten possible mechanisms for active PIs: publishing high novel papers, team size, number of different affiliations, probability to publish first-author, middle-author, and last author publications, maximum coauthor h index of each publication, research direction, reference overlapping, and hot topic (we define a paper covers a hot topic if a certain fraction of its MeSH terms all belong to the most frequently occurring MeSH terms, i.e., 10%, 50%, and 90%.). **(b)** The same as **a** but for the conservative removal as conducted in Fig. 3b. *** $p < .001$, ** $p < .05$, *$p < .1$; Error bars represent the standard errors, and are clustered at individual level.

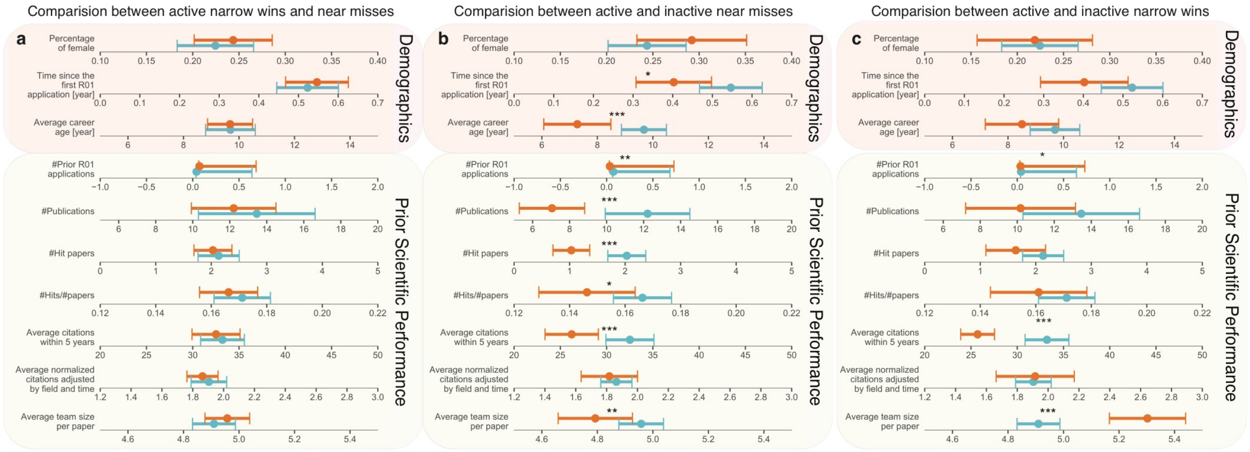

Supplementary Figure 29: Pre-treatment comparisons between the narrow-win and near-miss applicants in order to access the degree of the screening mechanism. (**a**) Pre-treatment feature comparisons between the near-miss (orange) and narrow-win (blue) group who are active in the next 10 years after treatment. We compared 10 different demographic and performance characteristics. The features are defined as follows (from top to bottom): 1) percentage of female applicants; 2) number of years since the first R01 application; 3) number of years since the first publication; 4) number of previous R01 applications; 5) number of publications prior to treatment; 6) number of prior papers that landed within the top 5% of citations within the same field and year; 7) probability of publishing a hit paper; 8) average citations papers received within 5 years of publication; 9) citations normalized by field and time11; and 10) average team size across prior papers. We see no significant difference between the two groups across any of the ten dimensions we measured. (**b**) The same as **a** but we compare active (blue) and inactive near misses (orange). (**c**) The same as **a** but the comparison between active (blue) and inactive narrow wins (orange). Error bar represents the 95% confidence interval. *** p < .001, ** p < .05, * p < .1.

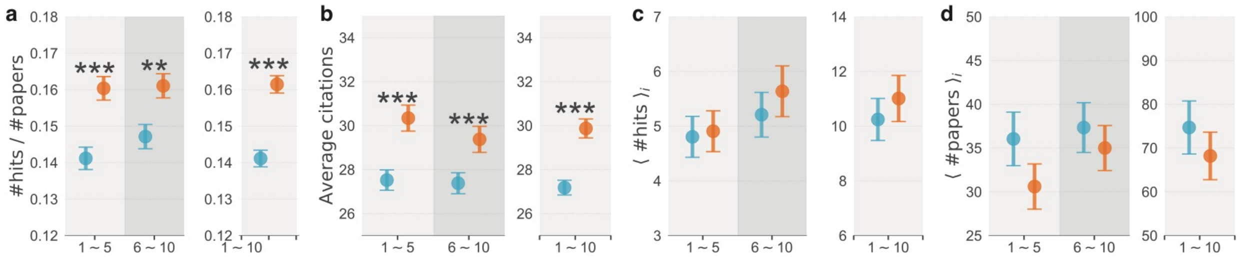

Supplementary Figure 30: Alternative ways to define less able PIs. Comparing narrow wins with near misses in terms of (**a**) hit rate per paper, (**b**) average citations within 5 years after publication (using data from 1990 to 2000), (**c**) hits per capita, and (**d**) number of publications per person. *** $p < .001$, ** $p < .05$, *$p < .1$; Error bar represents the standard error of the mean.

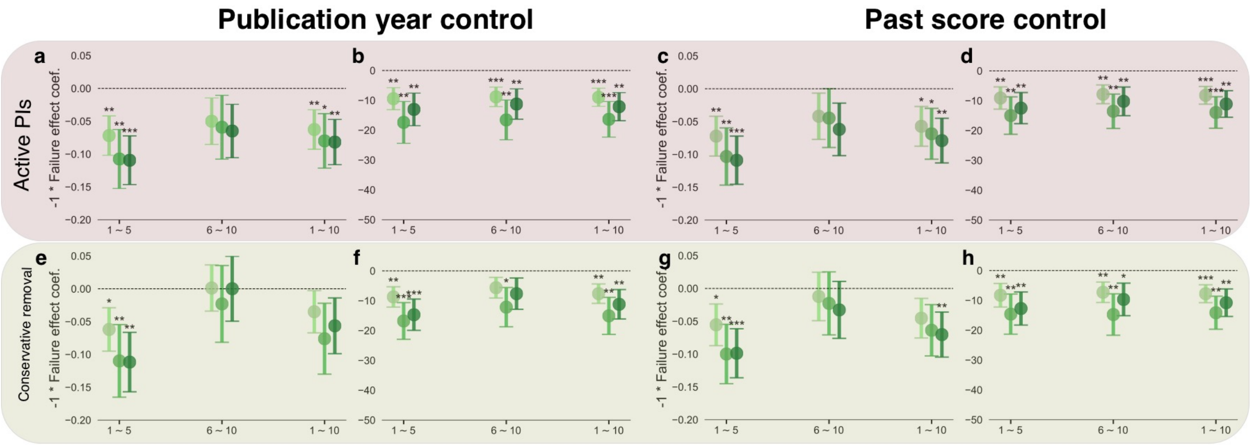

Supplementary Figure 31: The results from the fuzzy RDD estimation by adding publication time and PI past score fixed effects. (**a**) The effect of near miss on the probability to publish top 5% hit papers for applicants in the NIH system by adding publication time fixed effects as an additional control; (**b**) the effect of near miss on the average citations within 5 years after publication for applications in the NIH system by adding publication time fixed effects as an additional control; Here we use data from 1990 to 2000. (**c - d**) The same as **a – b** but with PI past score fixed effects; (**e - h**) The same as **a – d** but for the conservative removal. Here, we use three different sample size, i.e., 5-score around the cutoff, 10-score from the cutoff, and 25-score from the cutoff. Error bars represent the standard errors, and are clustered at individual PI level. *** p < .001, ** p < .05, *p < .1.

Supplementary References

1 Gerin, W. & Kapelewski, C. H. *Writing the NIH grant proposal: a step-by-step guide*. 2nd edn, (Sage Publications, 2011).

2 Sinatra, R., Wang, D., Deville, P., Song, C. & Barabasi, A. L. Quantifying the evolution of individual scientific impact. *Science* **354**, aaf5239 (2016).

3 Han, H., Zha, H. Y. & Giles, C. L. Name disambiguation spectral in author citations using a K-way clustering method. *Proceedings of the 5th Acm/Ieee Joint Conference on Digital Libraries, Proceedings*, 334-343 (2005).

4 Huang, J., Ertekin, S. & Giles, C. L. Efficient name disambiguation for large-scale databases. *Lect Notes Artif Int* **4213**, 536-544 (2006).

5 Liu, L. *et al.* Hot streaks in artistic, cultural, and scientific careers. *Nature* **559**, 396 (2018).

6 Treeratpituk, P. & Giles, C. L. Disambiguating Authors in Academic Publications using Random Forests. *Jcdl 09: Proceedings of the 2009 Acm/Ieee Joint Conference on Digital Libraries*, 39-48 (2009).

7 Tang, L. & Walsh, J. P. Bibliometric fingerprints: name disambiguation based on approximate structure equivalence of cognitive maps. *Scientometrics* **84**, 763-784 (2010).

8 Tekles, A. & Bornmann, L. Author name disambiguation of bibliometric data: A comparison of several unsupervised approaches. *arXiv preprint arXiv:1904.12746* (2019).

9 Caron, E. & van Eck, N. J. in *Proceedings of the 19th international conference on science and technology indicators.* 79-86 (CWTS-Leiden University Leiden).

10 Lin, J. C. Chinese names containing a non-Chinese given name. *Cataloging & classification quarterly* **9**, 69-81 (1988).

11 Jacob, B. A. & Lefgren, L. The impact of research grant funding on scientific productivity. *J Public Econ* **95**, 1168-1177 (2011).

12 Merton, R. K. The Matthew effect in science: The reward and communication systems of science are considered. *Science* **159**, 56-63 (1968).

13 Price, D. d. S. A general theory of bibliometric and other cumulative advantage processes. *Journal of the Association for Information Science and Technology* **27**, 292-306 (1976).

14 Barabasi, A. L. & Albert, R. Emergence of scaling in random networks. *Science* **286**, 509-512 (1999).

15 Barabási, A.-L. s. & Pósfai, M. r. *Network science*. (Cambridge University Press, 2016).

16 Zipf, G. K. *Human behavior and the principle of least effort: An introduction to human ecology*. (Ravenio Books, 2016).

17 Redner, S. Citation statistics from 110 years of Physical Review. *Phys Today* **58**, 49-54 (2005).

18 Newman, M. E. J. The first-mover advantage in scientific publication. *Epl* **86** (2009).

19 Allison, P. D., Long, J. S. & Krauze, T. K. Cumulative Advantage and Inequality in Science. *Am Sociol Rev* **47**, 615-625 (1982).

20 Price, D. J. D. Networks of Scientific Papers. *Science* **149**, 510-& (1965).

21 Azoulay, P., Stuart, T. & Wang, Y. Matthew: Effect or Fable? *Management Science* **60**, 92-109 (2014).

22 Petersen, A. M. *et al.* Reputation and impact in academic careers. *P Natl Acad Sci USA* **111**, 15316-15321 (2014).

23 Petersen, A. M., Jung, W. S., Yang, J. S. & Stanley, H. E. Quantitative and empirical demonstration of the Matthew effect in a study of career longevity. *P Natl Acad Sci USA* **108**, 18-23 (2011).

24 Cole, J. R. & Cole, S. Social stratification in science. *American Journal of Physics* **42**, 923-924 (1974).

25 Zuckerman, H. The sociology of science. *Handbook of Sociology (Newbury Park, Cali-fornia: Sage, 1988)* **547** (1988).

26 Melin, G. & Danell, R. The top eight percent: development of approved and rejected applicants for a prestigious grant in Sweden. *Science and Public Policy* **33**, 702-712 (2006).
27 Muchnik, L., Aral, S. & Taylor, S. J. Social Influence Bias: A Randomized Experiment. *Science* **341**, 647-651 (2013).
28 van de Rijt, A., Kang, S. M., Restivo, M. & Patil, A. Field experiments of success-breeds-success dynamics. *P Natl Acad Sci USA* **111**, 6934-6939 (2014).
29 Restivo, M. & van de Rijt, A. Experimental Study of Informal Rewards in Peer Production. *Plos One* **7**, e34358 (2012).
30 Atkinson, J. W. *An introduction to motivation*. (Van Nostrand, 1964).
31 Cole, J. R. & Cole, S. *Social stratification in science*. (University of Chicago Press, 1973).
32 Ganguli, I. Saving Soviet Science: The Impact of Grants When Government R& D Funding Disappears. *Am Econ J-Appl Econ* **9**, 165-201 (2017).
33 Bol, T., de Vaan, M. & van de Rijt, A. The Matthew effect in science funding. *P Natl Acad Sci USA* **115**, 4887-4890 (2018).
34 Heggeness, M. L., Ginther, D. K., Larenas, M. I. & Carter-Johnson, F. D. The Impact of Postdoctoral Fellowships on a Future Independent Career in Federally Funded Biomedical Research. (National Bureau of Economic Research, 2018).
35 Elton, E. J., Gruber, M. J. & Blake, C. R. Survivor Bias and Mutual Fund Performance. *The Review of Financial Studies* **9**, 1097-1120 (1996).
36 Brown, S. J., Goetzmann, W., Ibbotson, R. G. & Ross, S. A. Survivorship Bias in Performance Studies. *Rev Financ Stud* **5**, 553-580 (1992).
37 Sitkin, S. B. Learning through Failure - the Strategy of Small Losses. *Res Organ Behav* **14**, 231-266 (1992).
38 Edmondson, A. Psychological safety and learning behavior in work teams. *Admin Sci Quart* **44**, 350-383 (1999).
39 Taleb, N. N. *Antifragile: Things that gain from disorder*. (Random House Incorporated, 2012).
40 Kwiek, M. The European research elite: a cross-national study of highly productive academics in 11 countries. *High Educ* **71**, 379-397 (2016).
41 Burnette, J. L., O'Boyle, E. H., VanEpps, E. M., Pollack, J. M. & Finkel, E. J. Mind-Sets Matter: A Meta-Analytic Review of Implicit Theories and Self-Regulation. *Psychol Bull* **139**, 655-701 (2013).
42 Carver, C. S. & Scheier, M. *On the self-regulation of behavior*. (Cambridge University Press, 1998).
43 Radicchi, F., Fortunato, S. & Castellano, C. Universality of citation distributions: Toward an objective measure of scientific impact. *P Natl Acad Sci USA* **105**, 17268-17272 (2008).
44 Hutchins, B. I., Yuan, X., Anderson, J. M. & Santangelo, G. M. Relative Citation Ratio (RCR): A New Metric That Uses Citation Rates to Measure Influence at the Article Level. *Plos Biol* **14** (2016).
45 Waltman, L. *NIH's new citation metric: A step forward in quantifying scientific impact?*, <https://www.cwts.nl/blog?article=n-q2u294> (2015).
46 Powell, K. The Waiting Game. *Nature* **530**, 148-151, doi:DOI 10.1038/530148a (2016).
47 Bornmann, L., Marx, W. & Barth, A. The normalization of citation counts based on classification systems. *Publications* **1**, 78-86 (2013).
48 Bornmann, L. Scientific Peer Review. *Annu Rev Inform Sci* **45**, 199-245 (2011).
49 Ginther, D. K. *et al.* Race, Ethnicity, and NIH Research Awards. *Science* **333**, 1015-1019, doi:10.1126/science.1196783 (2011).
50 Ambekar, A., Ward, C., Mohammed, J., Male, S. & Skiena, S. in *Proceedings of the 15th ACM SIGKDD international conference on Knowledge Discovery and Data Mining.* 49-58 (ACM).
51 Weber, G. M. Identifying translational science within the triangle of biomedicine. *Journal of translational medicine* **11**, 126 (2013).

52 *IDENTIFYING TRANSLATION: iTrans*, <https://dpcpsi.nih.gov/sites/default/files/iTrans_one_pager 04172018.pdf> (2019).
53 Wang, J., Veugelers, R. & Stephan, P. Bias against novelty in science: A cautionary tale for users of bibliometric indicators. *Research Policy* **46**, 1416-1436 (2017).
54 Uzzi, B., Mukherjee, S., Stringer, M. & Jones, B. Atypical combinations and scientific impact. *Science* **342**, 468-472 (2013).
55 Wuchty, S., Jones, B. F. & Uzzi, B. The increasing dominance of teams in production of knowledge. *Science* **316**, 1036-1039 (2007).
56 Council, N. R. *Enhancing the effectiveness of team science*. (National Academies Press, 2015).
57 Zhang, C. T. A proposal for calculating weighted citations based on author rank. *Embo Rep* **10**, 416-417 (2009).
58 Wei, T. *et al.* Do scientists trace hot topics? *Sci Rep-Uk* **3**, 2207 (2013).
59 Lee, D. S. & Lemieux, T. Regression Discontinuity Designs in Economics. *J Econ Lit* **48**, 281-355 (2010).
60 Angrist, J. D. & Pischke, J. S. *Mostly harmless econometrics: an empiricist's companion*. (Princeton University Press, 2009).
