# Supplementary material for: Early-career setback and future career impact
Source: Nat Commun. 2019 Oct 1;10:4331. doi: 10.1038/s41467-019-12189-3 (PMC6773762; doi:10.1038/s41467-019-12189-3)
Supplement: Supplementary file 3 — Reporting Summary [file 41467_2019_12189_MOESM3_ESM.pdf]

nature research

Corresponding author(s): Dashun Wang

Last updated by author(s): Aug 10, 2019

Reporting Summary

Nature Research wishes to improve the reproducibility of the work that we publish. This form provides structure for consistency and transparency in reporting. For further information on Nature Research policies, see Authors & Referees and the Editorial Policy Checklist.

Statistics

For all statistical analyses, confirm that the following items are present in the figure legend, table legend, main text, or Methods section.

n/a	Confirmed	
☐	☒	The exact sample size (*n*) for each experimental group/condition, given as a discrete number and unit of measurement
☐	☒	A statement on whether measurements were taken from distinct samples or whether the same sample was measured repeatedly
☐	☒	The statistical test(s) used AND whether they are one- or two-sided<br>*Only common tests should be described solely by name; describe more complex techniques in the Methods section.*
☐	☒	A description of all covariates tested
☐	☒	A description of any assumptions or corrections, such as tests of normality and adjustment for multiple comparisons
☐	☒	A full description of the statistical parameters including central tendency (e.g. means) or other basic estimates (e.g. regression coefficient) AND variation (e.g. standard deviation) or associated estimates of uncertainty (e.g. confidence intervals)
☐	☒	For null hypothesis testing, the test statistic (e.g. *F*, *t*, *r*) with confidence intervals, effect sizes, degrees of freedom and *P* value noted<br>*Give P values as exact values whenever suitable.*
☒	☐	For Bayesian analysis, information on the choice of priors and Markov chain Monte Carlo settings
☒	☐	For hierarchical and complex designs, identification of the appropriate level for tests and full reporting of outcomes
☐	☒	Estimates of effect sizes (e.g. Cohen's *d*, Pearson's *r*), indicating how they were calculated

Our web collection on statistics for biologists contains articles on many of the points above.

Software and code

Policy information about availability of computer code

Data collection	The grant applications datasets were provided by the NIH. The Web of Science data was obtained through a purchase agreement with Thomson Reuters. Profiles of scientists were automatically collected from Google Scholar. Data collection was conducted using Python 2.7.
Data analysis	Data analyses were conducted using Python 2.7 and Stata 14.0.

For manuscripts utilizing custom algorithms or software that are central to the research but not yet described in published literature, software must be made available to editors/reviewers. We strongly encourage code deposition in a community repository (e.g. GitHub). See the Nature Research guidelines for submitting code & software for further information.

Data

Policy information about availability of data

All manuscripts must include a data availability statement. This statement should provide the following information, where applicable:

- Accession codes, unique identifiers, or web links for publicly available datasets
- A list of figures that have associated raw data
- A description of any restrictions on data availability

This paper makes use of restricted access data from the National Institutes of Health, protected by the Privacy Act of 1974 as amended (5 U.S.C. 552a). De-identified data necessary to reproduce all plots and statistical analyses are available at https://yang-wang.github.io/setback/. Those wishing to access the raw data may apply for access following the procedures outlined in the NIH Data Access Policy document available at http://report.nih.gov/pdf/DataAccessPolicy.pdf. The Web of Science data are available via Clarivate Analytics. The PubMed data are publicly available as discussed in the supplementary information.

Field-specific reporting

Please select the one below that is the best fit for your research. If you are not sure, read the appropriate sections before making your selection.

☐ Life sciences ☒ Behavioural & social sciences ☐ Ecological, evolutionary & environmental sciences

For a reference copy of the document with all sections, see nature.com/documents/nr-reporting-summary-flat.pdf

Behavioural & social sciences study design

All studies must disclose on these points even when the disclosure is negative.

Study description	This is a quantitative study of individual career histories based on pre-existing datasets
Research sample	We assembled our datasets from a range of different sources: (1) the U.S. National Institutes of Health (NIH) grant application data; (2) Thomson Reuters' Web of Science data (WoS); (3) the National Library of Medicine's PubMed database; and (4) Google Scholar (GS) profiles. The NIH data consist of 778,219 competing grant applications in total, supporting more than 170,000 research personnel across more than 2,500 U.S. institutions. The Web of Science data contains more than 46 million papers published from 1900 to 2015 by more than 20,000 different journals. We focus on all new competing grant applications from regular standing study sections between FY1990 and FY2005. Detailed selection criteria within a dataset are described in the supplementary information.
Sampling strategy	No statistical methods were used to predetermine sample size.
Data collection	This study is based on pre-existing datasets.
Timing	Web of Science and Google Scholar were collected in summer 2015. The NIH dataset was collected in summer 2016.
Data exclusions	The analysis has no data exclusions. Selection criteria within a dataset are described in the supplementary information.
Non-participation	There are no participants in this study.
Randomization	This is a data driven study, not a randomized experiment.

Reporting for specific materials, systems and methods

We require information from authors about some types of materials, experimental systems and methods used in many studies. Here, indicate whether each material, system or method listed is relevant to your study. If you are not sure if a list item applies to your research, read the appropriate section before selecting a response.

Materials & experimental systems

n/a	Involved in the study	
☒	☐	Antibodies
☒	☐	Eukaryotic cell lines
☒	☐	Palaeontology
☒	☐	Animals and other organisms
☒	☐	Human research participants
☒	☐	Clinical data

Methods

n/a	Involved in the study	
☒	☐	ChIP-seq
☒	☐	Flow cytometry
☒	☐	MRI-based neuroimaging
